# Versatility of Click Chemistry in Hydrogel Synthesis: From Molecular Strategies to Applications in Regenerative Medicine

**DOI:** 10.3390/gels12020127

**Published:** 2026-02-01

**Authors:** Domingo Cesar Carrascal-Hernández, Carlos David Grande-Tovar, Daniel Insuasty, Edgar Márquez, Maximiliano Mendez-Lopez

**Affiliations:** 1 Departamento de Química y Biología, Facultad de Ciencias Básicas, Universidad del Norte, Barranquilla 080020, Colombia; ebrazon@uninorte.edu.co (E.M.); maximilianom@uninorte.edu.co (M.M.-L.); 2 Grupo de Investigación de Fotoquímica y Fotobiología, Programa de Química, Universidad del Atlántico, Carrera 30 No. 8-49, Puerto Colombia 081007, Colombia; carlosgrande@mail.uniatlantico.edu.co

**Keywords:** click chemistry, tissue engineering, hydrogels, chitosan, regenerative medicine

## Abstract

Click chemistry is highly valued in the design of polymeric biomaterials due to its ability to generate complex structures and localized surface modifications. However, prominent mechanisms in click chemistry, such as copper-catalyzed azide-alkyne cycloaddition (CuAAC), are inefficient for the synthesis and/or modification of biomaterials because they present significant limitations for in vivo applications. The presence of residual copper in the material is toxic and requires extensive purification, increasing production costs and hindering scalability and availability for in vivo applications. To overcome these limitations and ensure the safety and biocompatibility of materials, biorthogonal reactions such as strain-promoted azide-alkyne cycloaddition (SPAAC) have been developed. Thiol-ene/thiol-yne and Diels–Alder mechanisms are also relevant for the formation of robust polymer networks with specific characteristics and attractive advantages for generating biocompatible materials. These reactions not only improve cell integration and reduce fibrosis in in vivo applications but also enable the creation of functional structures for tissue regeneration. This review provides a comprehensive analysis of advances in the synthesis of biomaterials for tissue regeneration using hydrogels designed via click chemistry, as well as the various mechanisms and structural considerations.

## 1. Introduction

Polymers represent a versatile class of materials with a constantly evolving structural diversity, capable of interacting harmoniously with physiological and biological systems [1,2,3]. In recent decades, there has been a growing interest in the development and application of polymers, whether synthetic, natural, or hybrid, in the medical field. Notable examples include polylactic acid (PLA), chitin/chitosan (CS), fibrin, collagen, polyglycolic acid (PGA), the copolymer poly(lactic-co-glycolic acid) (PLGA), and polyalkanoates such as poly(hydroxybutyrate) (PHA), among others [4]. Three-dimensional structures with adjustable porosity have been explored using these polymers, enabling the diffusion of various biomolecules and cell adhesion, a critical aspect for the material’s viability [5]. However, it is essential to note that their direct application as an in vivo graft to promote soft tissue regeneration faces significant limitations. For example, these materials have mechanical and biological limitations that do not meet the clinical requirements for material viability. These limitations can be overcome with various chemical functionalizations of the material, but their functionalization is complex and requires highly sophisticated approaches [6]. Nevertheless, such functionalization requires highly targeted chemical modifications, a process that must avoid the formation of undesirable byproducts that could compromise the biomaterial’s biocompatibility or structural stability [7,8].

Hydrogels constitute a diverse and functional class of biomaterials with broad potential in biomedical applications. Their structure is based on three-dimensional networks of hydrophilic chains forming a highly porous matrix capable of retaining large volumes of water or physiological solutions [9]. Several structural features in these materials facilitate chemical reactions that enable modulation of their porosity. Prominent examples include hydrogels based on fibrogen grafted with polyethylene glycol (PEG), which improve the material’s porosity, promoting the adhesion and growth of breast cancer cells in in vitro cultures, thereby increasing cell availability for clinical trials [10]. Furthermore, its structure exhibits some similarity to extracellular matrices, which improves its performance in physiological media compared to other traditional materials without grafts [11].

Synthetic, natural, and hybrid polymers (mixtures of synthetic and natural polymers) have been reported to serve as grafts in these materials [12,13]. For example, collagen crosslinking to form hydrogels has been reported due to its excellent biocompatibility with soft and connective tissues; it has also been shown to promote cell adhesion [14]. Similarly, the physical crosslinking of alginate using calcium ions (Ca^2+^) has been reported to promote cell encapsulation and the controlled release of drugs and biomolecules [15]. Poly(N-isopropylacrylamide) (PNIPAAm)-based hydrogels exhibit thermosensitive properties with reversible phase transitions in response to temperature changes. These characteristics are highly valued for in vivo applications because they enable modulation of structural and mechanical properties through thermal therapies in implants [16].

On the other hand, photopolymerization of water-soluble monomers could be an attractive synthesis method for generating these materials, as it is appealing for in vivo applications of injectable hydrogels. However, this approach does not provide controlled crosslinking of the material. Furthermore, these polymerization approaches proceed via radical mechanisms, which pose critical limitations for in vivo applications and yield structures with heterogeneity, poor mechanical properties, and poorly defined molecular architectures [17]. This lack of homogeneity and chemical selectivity compromises the reproducibility and viability of hydrogels in demanding clinical environments where structural precision is a critical factor [18]. To address these limitations, click chemistry has emerged as a highly efficient and selective synthetic strategy for constructing hydrogels with well-defined, sophisticated molecular architectures [19]. This approach allows for the concerted crosslinking of polymers under mild conditions, with high specificity and without generating undesirable byproducts [20,21]. Notable examples include hydrogels based on copper-catalyzed azide-alkyne cycloaddition (CuAAC), which have been used to form polymer networks with spatial and temporal control [22,23]; hydrogels derived from thiol-ene reactions that allow rapid and biocompatible cross-linking [24], systems based on reversible Diels–Alder reactions, which offer self-repairing and adaptive properties [25], and strain-promoted azide–alkyne cycloaddition (SPAAC) reactions, which are exceptional for in vivo applications since they avoid the use of metal catalysts and high temperatures, thus preventing the cytotoxicity of metals in the CuAAC mechanism and the thermal damage of the classic Huisgen mechanism, and allowing the reaction kinetics to be optimized without the use of catalysts. For example, SPAAC reactions, such as the elimination of vinyl/enol triflates, led to the synthesis of monofluorinated cyclooctines (MOFO) and difluorinated cyclooctine (DIFO), in which fluorine atoms are introduced near alkyne groups, thereby reducing the LUMO level and improving interaction with the HOMO of the azide [26]. Other variants of SPAAC consist of cyclopropenone photodecarboxylation mechanisms, sulfoxide–magnesium exchange, co-complex decomplexation, silylated allene rearrangement, and carbene-mediated strategies [27].

The relevance of click chemistry in this context lies in its ability to overcome the structural and functional barriers that limit the performance of conventional hydrogels. By offering a modular, predictable, and highly controlled platform, this strategy not only improves the chemical and mechanical uniformity of the material but also enables the targeted incorporation of bioactive motifs, molecular sensors, or therapeutic agents [28]. In this regard, hydrogels obtained through click chemistry not only exhibit superior properties in terms of stability, functionality, and biocompatibility but also open new possibilities for the design of smart systems in regenerative medicine, controlled drug delivery, and tissue bioengineering. For this reason, this review article thoroughly examines the synthesis of hydrogels through concerted reactions and the click-chemistry approach, from molecular design to multifunctional properties and potential applications in biomedical engineering.

## 2. Critical Factors in Applying Click Chemistry for Material Design: Reaction Kinetics, Conditions, and Structural Stability

**Figure 1:**
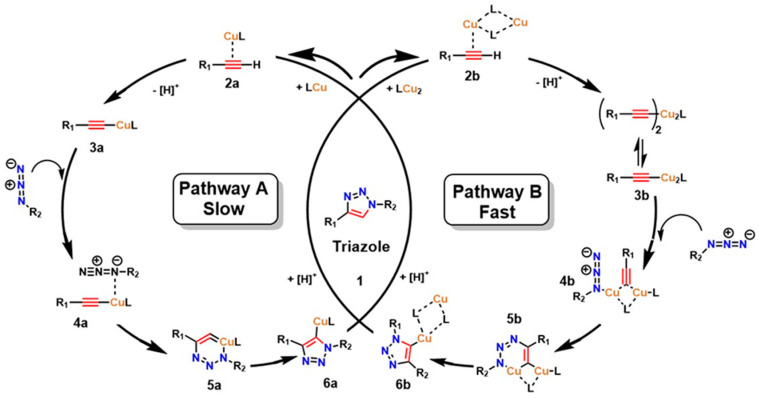
Catalytic cycle of the 1,3−dipolar cycloaddition reactions between azides and terminal alkynes, known as copper−catalyzed azide–alkyne cycloaddition (CuAAC). Adapted from Fantoni et al. [36].

The interest in leveraging the diverse possibilities of molecular assembly in a highly selective manner as a tool for designing and expanding the structural diversity of functional compounds led to the development of what is now known as “click chemistry,” introduced by Sharpless et al. [29]. This strategy has revolutionized combinatorial synthesis by enabling the formation of new covalent bonds through highly selective, efficient, and rapid reactions [30]. A prominent example is the 1,3-dipolar cycloaddition between azides and terminal alkynes, known as the copper-catalyzed azide–alkyne cycloaddition (CuAAC), which forms new carbon–heteroatom bonds in a concerted manner, thereby facilitating precise linkage between diverse molecules through coupling agents [31], as shown in Figure 1. Kinetic studies and calculations based on density functional theory (DFT) have confirmed that two prevalent mechanisms exist in CuAAC for triazole formation: a slow process catalyzed by a mononuclear Cu^+^ species (**Path A**) and a kinetically favored route promoted by the formation of a dinuclear Cu catalyst (**Path B**) [32,33,34].

The substitution step by a donor ligand on the organic alkyne at the Cu(I) metal center defines both mechanistic pathways, resulting in a highly stabilized π complex (as shown in species **2a** and **2b**). Electronic rearrangement then occurs around Cu(I), establishing the necessary activation conditions at the triple bond of the organic alkyne, thereby favoring the reaction’s advancement. In this sense, the π interaction between the alkyne triple bond and the metal center imposes a determining step in the electron density distribution, which has a decisive effect on the efficiency and selectivity of the overall mechanism [32]. From a thermodynamic point of view, the reaction medium significantly influences this step. That is, in organic media, the initial substitution shows a slightly endothermic character, with an energy of around 0.6 kcal/mol. In contrast, in aqueous media, the process is more exothermic, with an energy of around 11.7 kcal/mol [32]. This marked difference not only demonstrates the process’s efficiency in aqueous media but also supports experimental data reporting a higher reaction rate in aqueous media [35]. The coordination of the alkyne to the metal center establishes the metal–alkyne adduct, inducing a significant decrease in the alkyne’s pKa (by nearly 10 units), which facilitates its deprotonation and the subsequent formation of the Cu–acetylide complex (species **3a** and **3b**), even under aqueous conditions. This electronic and acid–base adjustment constitutes a critical point for substrate activation [36].

Furthermore, after the rapid formation of the Cu-acetylide complex, the mechanism proceeds with the coordination of the azide to the Cu(I) core or to the second metal center in the dinuclear pathway (species **4a** and **4b**). This event drives the endothermic addition of the terminal nitrogen (-N_3_) of the azide to the C-2 carbon of the acetylide, resulting in a transient Cu(III) metallacycle (species **5a** and **5b**). The intervention of Cu is decisive at this stage, as it facilitates the construction of the C–N bond and significantly reduces the activation energy (Ea) compared to the uncatalyzed reaction, from values close to 26 kcal/mol to ranges of 15–19 kcal/mol, depending on the nature of the substituents coordinated to the metal center. The cycle concludes with the formation or contraction of the ring that yields the triazoline-Cu intermediate. This, in turn, undergoes proteolysis, releasing the triazoline ring as a stable product and regenerating the initial catalyst (as shown in species **6a** and **6b**). This step highlights the efficiency of Cu as a catalyst in activation and electronic reorganization, reinforcing its relevance in 1,3-dipolar cycloaddition reactions [36].

This mechanism has boosted developments in the synthesis of conjugated materials and led to significant advances in combinatorial synthesis, expanding molecular diversity in the synthesis of new materials [37]. For example, this mechanism allowed the synthesis of copolymers with adjustable chemical characteristics, overcoming the structural limitations of traditional polymers. A notable case is the synthesis of diethylphosphoryldithioformate and pyridine-2-yldithioformate with coupling agents of 2, 3, and/or 4 alkyne groups, which has allowed the synthesis of three- and four-armed polystyrene copolymers, which exhibit mechanical and thermal properties superior to traditional materials [38]. Similarly, this approach has overcome the limitations of conventional reactions, offering more efficient, reproducible, and adaptable synthetic routes for different environments [39]. However, the presence of residual Cu in the materials makes this approach unsuitable for synthesizing biocompatible materials for medical applications, as Cu toxicity triggers inflammatory responses in tissues [35].

### 2.1. Regioselectivity and Molecular Diversity in Azide-Alkyne Cycloaddition in Materials Design: Huisgen vs. CuAAC

It has been demonstrated that Cu (generally in the +1 oxidation state), present in the synthesis of triazoles through the CuAAC mechanism, in the synthesis or functionalization of materials, can form transient complexes with various functional groups present in polymer structures of interest in medicine [40]. That is, Cu(I) can coordinate with electron-donating functional groups such as amides, phosphates, hydroxyls, and carboxylates, which are common in biomaterials such as chitosan, collagen, and other materials of interest in medicine. However, the presence of Cu that does not form stable complexes with the materials can compete for high-affinity metal-binding sites on enzymes and proteins, leading to enzyme deactivation and structural damage to proteins [41]. In addition, Cu catalyzes Fenton-type reactions, generating reactive oxygen species (ROS) that alter DNA, lipids, and proteins and can induce apoptosis and necrosis [42].

That is, Li et al. studied the uptake of residual Cu in umbilical cord endothelial cells (HUVEC) using inductively coupled plasma mass spectrometry (ICP-MS), where it was determined that a concentration of 52 ± 3 μM generates inflammatory responses in tissues, charged species that give rise to ROS, and Cu interacts with other charged biomolecules, affecting their functioning. In this sense, controlling residual Cu in materials synthesized or functionalized via CuAAC is very challenging, which is prohibitive for the synthesis of biofunctional materials [43]. However, Huisgen’s classic method (1,3-dipolar cycloaddition) does not require metal catalysts for the synthesis of triazoles in material functionalization, thereby avoiding metal-related cytotoxicity. Still, it requires high temperatures (100–150 °C) that cause thermal damage and low selectivity (1.4 and 1.5), making it incompatible with sensitive materials and biological systems, which is problematic for the functionalization of demanding materials [44].

**Figure 2:**
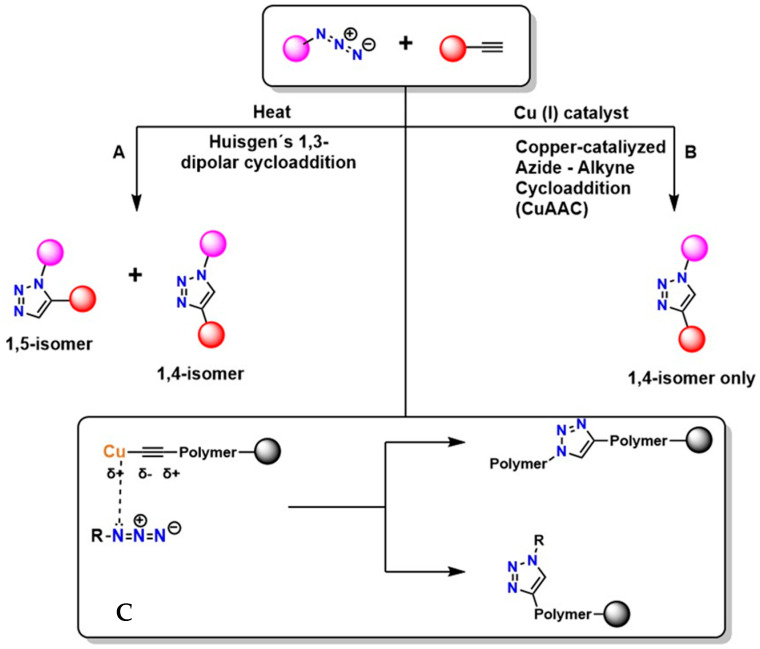
Huisgen cycloaddition mechanism (**A**), copper−catalyzed azide−alkyne mechanism (**B**), and peptidotriazoles (**C**).

Figure 2 shows two main pathways for the reaction between an azide (R–N_3_^+^) and a terminal alkyne, highlighting two principal mechanisms: thermal cycloaddition and Cu-catalyzed cycloaddition [45]. In Figure 2A, the Huisgen 1,3-dipolar cycloaddition occurs, generating a mixture of isomers: the 1,4-disubstituted and the 1,5-disubstituted. This process is not regioselective and requires high temperatures, which limits its applicability in sensitive systems. Furthermore, according to Breugst et al., this reaction exhibits slow kinetics and lacks regioselective control [46].

In Figure 2B, CuAAC is shown, considered the model and starting point of click chemistry. The presence of Cu(I) provides regioselectivity, whereas the Huisgen 1,3-dipolar cycloaddition does not, generating the 1,4-disubstituted isomer exclusively under mild conditions and with high efficiency [47]. The mechanism of this reaction involves Cu coordination to the alkyne, which polarizes the triple bond and facilitates the formation of the triazole [48]. This reaction is highly valued in the synthesis of functional polymers due to its high yield [49]. In Figure 2C, the triazole is shown incorporated into the polymer structure, where it can act as a crosslinking agent or as a functionalization point with an R group of specific properties [50]. This process is widely used in the synthesis of advanced materials and in the surface modification of various substrates due to its high efficiency and compatibility with diverse environments. However, even with improvements in regioselectivity, the presence of Cu remains problematic because of its cytotoxic effects [51].

On the other hand, alternative reactions have been reported that avoid the use of Cu, thereby expanding the possibilities for synthesizing biocompatible materials. Among these alternatives are nucleophilic ring-opening reactions of epoxides and aziridines [52], hydrazone formation [53], and the synthesis of non-aldolic heterocycles [54]. In addition, strain-promoted azide–alkyne cycloaddition (SPAAC), better known as the copper-free cycloaddition reaction, occurs through chemical species such as 5-methylcyclooct-1-yne with an azide group to generate 1,6-dimethyl-4,5,6,7,8,9-hexahydro-1H-cycloocta[d][1,2,3]triazole. This mechanism has been employed for highly efficient and exclusive bioorthogonal reactions, as well as reactions based on cyclooctynes such as dibenzocyclooctynes (DBCO) and azides to form the [3 + 2] ring [55,56]. Furthermore, reactions using alkynes such as trans-cyclooctyne (TCO), dibenzoazacyclooctyne (DIBAC), biarylazacyclooctinone (BARAC), and bicyclo [6.1.0]nonyne (BCN), which exploit enthalpic energy release to drive cycloaddition, are relevant for the design of biofunctional materials [57].

These reactions avoid the use of the Cu catalyst employed in the CuAAC method, which can generate oxidative processes and undesirable side reactions in biological systems [58]. However, alternative reactions, such as SPAAC, are up to 100 times slower than CuAAC [59]. In addition, the use of bulky rings imposes steric constraints, which are problematic for their incorporation into biomolecules or highly crosslinked materials [60].

Moreover, in the last decade, significant advances have been made in click-type reactions, attracting attention from researchers across various medical fields, including the development of hydrogels for implant coatings and soft tissue regeneration [61]. For example, Table 1 reports several reactions in hydrogel synthesis, such as thiol-based reactions using (1R,4S)-5-methylbicyclo [2.2.2] oct-2-ene, which are initiated by UV/Vis under physiological conditions, offering specific control during gelation. This control is highly complex with traditional thermal initiators. In addition, the Diels–Alder (DA) reaction constitutes a thermally reversible cycloaddition that enables the formation of hydrogels with tunable viscoelastic and self-healing properties, with gelation times ranging from 1 to 10 min. This is attractive for medical applications because free-radical polymerization enables crosslinking without glutaraldehyde, which can compromise cellular viability [62]. Likewise, oxime-based reactions have enabled the development of hydrogels containing heteroatom groups (N and O), thereby improving their performance in physiological systems and enhancing biocompatibility.

**Table 1:** Click-type reactions: kinetic parameters and relevance in regenerative medicine applications.

Reaction	Mechanism	$\mathrm{Rate}\;\mathrm{Constant}\;(M^{-1}s^{-1})$	Activation Energy (kJ/mol)	Advantages	Disadvantages	Ref.
CuAAC	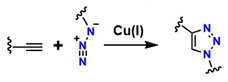	$10^{2}-10^{3}\;$	~40	High efficiency for the synthesis of materials with adjustable mechanical properties in materials science.	Requires metallic catalysts such as copper (Cu) or ruthenium (Ru), which affect the material’s cell viability for medical applications.	[34]
SPAAC	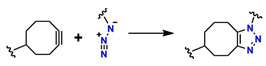	$10^{-2}-10^{0}\;$	~60	It is metal-free, which is excellent for in vivo applications, and it is bioorthogonal, so it does not interfere with biomolecules.	It has slow kinetics, which prolongs the material’s gelation time; it requires strained alkynes such as cyclooctynes, which are expensive and not readily available.	[63]
DA	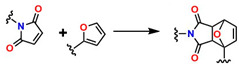	$10^{3}-10^{5}$	~20	It does not require catalysts that affect cell viability; its rapid kinetics make it ideal for instantaneous crosslinking of hydrogels; and it exhibits high tolerance in physiological media.	It requires expensive reagents such as tetrazines and dienophiles, and it is susceptible to oxidation.	[64]
Thiol-ene	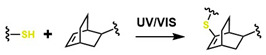	$10^{0}-10^{2}$	30–50	It is versatile for the design and synthesis of a wide variety of polymers with adjustable characteristics and features, and it enables controlled photopolymerization.	Because it employs radical reactions, this could cause tissue damage if used in situ; it requires UV as an initiator; it is sensitive to oxygen; and it exhibits disulfide exchange degradation effects in physiological media.	[65]
Thiol-ene	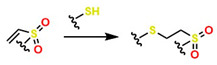	$10^{1}-10^{3}$	25–40	Its kinetics are faster than thiolene, it generates more cross-linked networks, conferring better mechanical behavior, and it exhibits multiple functionalization.	It has radical limitations, like the thiolene mechanism; requires UV light as an initiator; and its reagents are costly.	[66]
Oximas	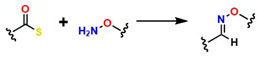	$10^{-3}-10^{-1}$	50–70	It is bioorthogonal and metal-free, generating structures with highly stable bonds compatible with physiological systems.	It exhibits slow kinetics, strong pH dependence, and requires mild catalysts such as aniline.	[67]

In polymer systems, post-polymerization modifications can be performed to functionalize polymers with alkyne groups. For example, radical polymerization, ring-opening polymerization (ROP), and addition polymerization are standard and suitable methods that allow a uniform distribution of alkyne groups within the polymer structure [68]. Among the most common post-polymerization modifications are nucleophilic substitution reactions (e.g., conversion of halides into alkynes using acetylenes), Sonogashira coupling (to introduce aromatic alkynes), and esterification or amidation with derivatives of terminal alkynes [50]. However, although click-type reactions such as CuAAC, SPAAC, DA, and thiol–ene/thiol–yne are attractive for intra- and intermolecular modifications (including surface modifications of materials), it is essential to consider the reaction rate (k) and activation energy associated with these reactions, as these factors can determine the efficiency or suitability of the synthesized materials [50]. For example, Table 1 reports that CuAAC is much faster than reactions such as SPAAC. Still, the latter has a fundamental advantage: it avoids metal catalysts that compromise material biocompatibility. Additionally, photochemically initiated reactions such as thiol–ene exhibit rapid kinetics but depend on radical formation and the absence of oxygen [65].

### 2.2. Molecular Insights into Click Chemistry Mechanisms for the Rational Design of Polymers with Tunable Functional Properties

From the perspectives of computational chemistry and molecular orbital theory, click-type reactions (such as the 1,3-dipolar cycloaddition between azides and alkynes) can be understood in greater depth by analyzing the frontier orbitals: the HOMO (Highest Occupied Molecular Orbital) and the LUMO (Lowest Unoccupied Molecular Orbital). These orbitals are involved in molecular recognition and represent the regions of highest electronic probability for charge transfer between reactive species; their interaction determines the feasibility and selectivity of the reaction [69].

In the case of CuAAC, the HOMO of the terminal alkyne interacts with the LUMO of the azide, facilitating the highly regioselective formation of the triazole ring [70]. The presence of the Cu catalyst stabilizes the intermediate complex. It lowers the activation energy (Ea), enabling the reaction to proceed under mild conditions, even in aqueous media and at room temperature [71]. The symmetry can explain the efficiency of this reaction and overlap between the alkyne’s π orbitals (HOMO) and the azide’s vacant orbitals (LUMO), leading to a concerted, symmetric transition state. This type of orbital interaction is consistent with Fukui’s frontier orbital theory, which states that chemical reactivity can be predicted based on the energetic and spatial proximity between the HOMO and LUMO of the reactants [72].

In this context, reactions between terminal alkynes and azides, catalyzed by Cu, proceed through an initial complexation between the terminal alkyne and Cu^+^. Moreover, the chemical nature of the alkynes determines the reaction rate. Electron-deficient alkynes (such as iodoalkynes) react with azides to form C–N bonds in a single step [73,74]. In other cases, C–N bond formation occurs sequentially: first, the azide binds to Cu^+^ through the nitrogen atom, followed by the attack of the alkyne carbon on a distal nitrogen atom. Several computational studies have demonstrated the regioselectivity of these reactions through the catalytic effect of Cu^+^ on the transition state energy [75]. Table 2 presents ten of the most relevant click-type reactions used in the synthesis of defined regioisomers. These transformations are characterized by high selectivity and efficiency, minimizing the formation of undesired mixtures under appropriate conditions. This level of control has been crucial for the development of clinically impactful molecules. An example of this is shown in reactions 1, 2, and 5 in Table 2. These compounds have served as the basis for the development of drugs now approved by the Food and Drug Administration (FDA), including antifungals such as fluconazole, voriconazole, posaconazole, and itraconazole, which are widely used in the treatment of systemic infections. Similarly, this strategy has driven the synthesis of compounds with antitumor activity, such as carboxamidotriazole (CAI) and mubritinib, demonstrating the versatility of the triazole nucleus in the rational design of medicines [76].

**Table 2:** Sample of click-type reactions in the synthesis of regioselective compounds for pharmaceuticals.

Entry	Compound	Reagents	Conditions	Product	Ref.
1	monosubstituted 1,2,3-triazoles	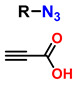 (1.5 eq.)	0.2 eq. CuI 0.4 eq. Na ascorbate 0.5 eq. DBU DMF 60 °C 3–24 h	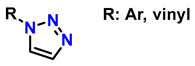	[77]
2	1,2,3-triazoles-4-monosubstituted	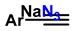 (1.5 eq.)	5 mol-% CuSO_4_ 5 mol-% (BimH)_3_ 0.25 eq. Sodium ascorbate	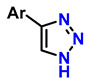	[78]
3	1-monosubstituted aryl 1,2,3-triazole	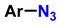 **CaC_2_**	0.3 eq. CuI 0.3 eq. Na ascorbate MeCN/H_2_O (2:1) r.t. 2–20 h	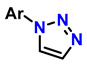	[79]
4	1,2,3-Monosubstituted triazoles CuI/ET_3_N	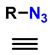 (1 atm)	0.1 eq. CuI 0.4 eq. ET_3_N DMSO r.t. 24 h	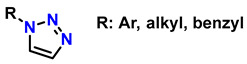	[80]
5	Highly regioselective synthesis of triazoles in water	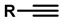 (1.1 eq.) 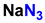 (1.2 eq.) 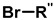	CuAl_2_O_4_ NPs (5 mg/mmol Br-R″)	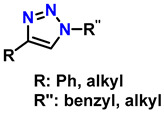	[81]
6	Tandem catalysis: from alkynoic acids and aryl iodides to 1,2,3-triazoles	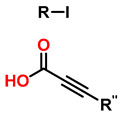	1.5 eq. NaN_3_, 0.2 eq. L-proline 0.1 eq. CuSO_4_·5H_2_O 0.2 eq. Na ascorbate, 1.2 eq. K_2_CO_3_ DMSO/H_2_O (9:1) 65 °C 20–24 h	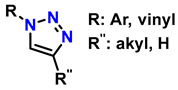	[82]
7	Self-assembly of copper sulfate and a poly(imidazol-acrylamide) amphiphile	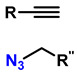	0.25 mol-% catalyst 0.1 eq. Na ascorbate H_2_O/tBuOH (3:1) 50 °C 1.5 h	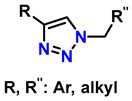	[83]
8	Synthesis of 5-alkyl-1,2,3-triazoles	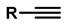 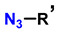 (1.5 eq.) 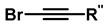 (2 eq.)	0.2 eq. CuI 2 eq. LiOtBu MS 4 Å DCE, r.t., 12 h	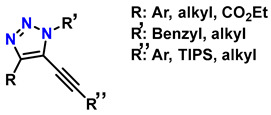	[84]
9	1,2,3-triazole derivatives via oxidative cycloaddition [3 + 2]	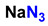 (4 eq.) 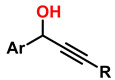	C(+)|SS(−), 11 mA (undivided cell) 0.7 eq. Bu_4_NI MeCN, r.t., 10 h	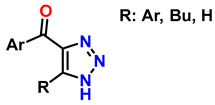	[85]
10	Synthesis of poly-lysubstituted compounds from 6-[(1H-1,2,3-triazol-1-yl)methyl]uracils	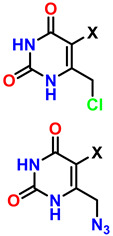	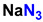 (1.05 eq.) DMF r.t., 2 h	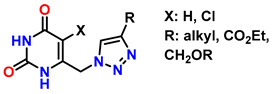	[86]

## 3. Integration of Click-Type Reactions in the Synthesis of Polymeric Materials

**Figure 3:**
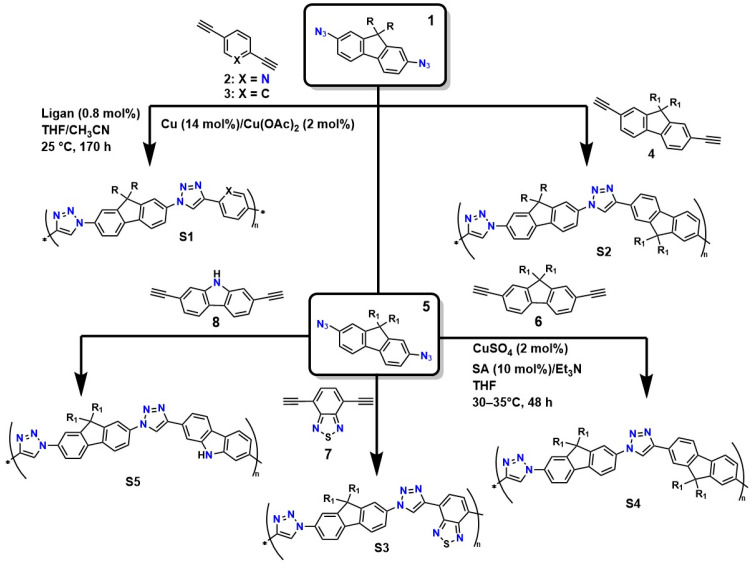
Synthesis of polymers with specific characteristics through concerted reactions.

The structural precision and specific characteristics required for designing advanced materials, such as hydrogels used in medical applications (particularly in regenerative medicine), demand highly sophisticated crosslinking methods [87]. In this context, click-type reactions have emerged as a versatile and powerful tool, offering a set of reactions governed by fundamental principles: thermodynamic parameters that ensure nearly quantitative yields, outstanding regio and stereoselectivity, orthogonality toward a wide range of functional groups, and compatibility with benign aqueous conditions [88]. This approach enables the rational design of polymer networks with tunable pore sizes, controlled crosslinking densities, and predefined viscoelastic properties, achieving a level of control unattainable through ionic crosslinking or conventional free-radical processes [89]. Figure 3 illustrates various routes for the synthesis of linear poly(triazoles) via the polymerization of 2,7-diazidofluorene with aromatic diynes, catalyzed by copper (compounds **2**–**4**). It also shows the preparation of soluble and π-conjugated poly(triazoles) through click-type polymerization (approximate yield of 90%) of 2,7-diazido-9,9-dioctylfluorene (**5**) with 2,7-diethynyl-9,9-dioctylfluorene (**6**), 4,7-diethynylbenzothiadiazole (**7**), and 2,7-diethynylcarbazole (**8**).

Van Steenis et al. demonstrated that the copolymers obtained via the copper-catalyzed process (**S1** and **S2**) had molecular weights near 25 kDa (PDI = 1.9). Under the same synthesis conditions, copolymers **S3**, **S4**, and **S5** reached molecular weights of up to 327 kDa (PDI = 1.21), 8 kDa (PDI = 1.61), and ∼6 kDa (PDI = 1.38), respectively [90]. The authors showed that the use of copper as a catalyst offers high efficiency and selectivity: it enables the production of polymers with high molecular weights and more controlled distributions (lower PDI), resulting in more homogeneous polymer networks [91]. Additionally, these systems are well compatible under mild conditions: the reaction occurs in aqueous media or benign solvents, reducing the risk of thermal degradation, and the method’s reproducibility makes it attractive for large-scale synthesis. However, it also has disadvantages, such as the presence of copper residues, which can be toxic and require additional purification steps for biomedical applications. Furthermore, strict control of ligands and conditions is necessary to avoid side reactions [91].

However, the synthetic origin of these polymeric structures can make them resistant to degradation in physiological systems, potentially interfering with soft tissue regeneration and leading to morbidity. Additionally, the presence of residual Cu is problematic for the application of these systems as implant coatings or carriers for the controlled release of biomolecules and drugs, due to Cu’s toxicity, which triggers inflammatory responses and generates reactive oxygen species (ROS) that affect various cellular processes [92]. On the other hand, alternative methods that avoid the use of Cu, such as the SPAAC method, eliminate the risk of contamination and inflammation caused by residual Cu but sacrifice efficiency and structural control, resulting in polymers with lower molecular weight and higher dispersity, which limits their mechanical and functional performance [93].

### 3.1. Design of Polymer Networks Based on Click Chemistry: Balance Between Rigidity and Bioactivity in Hydrogels

Moreover, the relationship between material crosslinking and hydrogel formation is fundamental to the biological functionality of hydrogels for regenerative medicine applications. The crosslinking density of materials is related to the rate of click reactions, which in turn influences properties such as the elastic modulus and, ultimately, cell adhesion. Hydrogels synthesized with a high density of triazole bonds exhibit high structural rigidity, which limits cell diffusion and proliferation and promotes pro-inflammatory (M1) macrophage polarization [94]. In contrast, hydrogels based on reversible mechanisms, such as Diels–Alder (DA), favor the formation of flexible networks that mimic the softness of native tissues, thereby facilitating angiogenesis [95]. Additionally, the structure and chemical characteristics of triazole rings condition the degradability kinetics of hydrogels under physiological conditions, which is essential for hydrogel implantation. However, triazole rings are highly stable to hydrolysis and oxidation, which is problematic in their application as grafts in highly invasive surgical procedures, as this affects tissue growth and may lead to fibrosis [29]. Conversely, hydrogels based on thiol–ene networks exhibit disulfide exchange behavior, offering gradual degradation in physiological environments and enabling the release of bioactive molecules [29].

For example, Table 3 reports successful cases of material modifications using click chemistry in soft tissue regeneration, where the SPAAC approach has been highly valued for collagen functionalization to promote localized cell adhesion and reconstruct corneal defects caused by injuries or surgical interventions. Other well-accepted methods include the inverse electron-demand Diels–Alder (IEDDA) reaction (a key reaction within bioorthogonal chemistry with applications in regenerative medicine), which is a variant of the classical Diels–Alder reaction but with inverted electronic demand: the diene is electron-poor (typically a heterodiene such as a tetrazine), and the dienophile is electron-rich (for example, an activated alkene or a norbornene) [96,97,98]. Additionally, the thiol–ene method, which involves the interaction between a thiol group (-SH) and a carbon–carbon double bond (C=C), is considered a click-type reaction since it meets the characteristics of click reactions: high efficiency, mild conditions, high selectivity, and formation of stable products [61,99,100].

**Table 3:** Strategies and Applications in Biomaterial Synthesis and Regenerative Therapies Using Click Chemistry.

Reaction	Reagents	Feature	Application	Advantages	Disadvantages	Ref.
SPAAC	Collagen	Cell fixation	Corneal and soft tissue defects	Re-epithelialization of soft tissues. For example, corneal tissue.	High costs that hinder scalability. Its kinetics are slower than those of the CuAAC pathway. Alkynes are bulky, which can alter collagen conformation and affect fibrillar organization. Although copper is not used, some tensional	[101]
	Hyaluronic acid Collagen	In situ cross-linking	Soft tissue defects	Good biocompatibility and adaptation to physiological systems, and they promote a microenvironment conducive to cell growth.	Alkynes are bulky, which can alter collagen conformation and affect fibrillar organization. Tensile alkynes (such as DBCO ^a^, BCN ^b^) are expensive, and their synthesis is complex.	[102]
	Hyaluronic acid Polyethylene glycol	Controlled release of peptides	Skin defects	Advantages: Re-epithelialization, collagen deposition, and localized microvessel formation and PCS ^c^.	Chemical modifications can affect the biological activity of hyaluronic acid or polyethylene glycol if not performed carefully. Despite their biocompatibility, these materials have lower mechanical strength, which may limit their use in applications requiring prolonged structural support.	[103]
	Metacryloyl gelatin	Self-adhesive	Nerve damage	Advantages: Nerve regeneration with faster conduction velocity and shorter latency.	Relatively low mechanical strength for applications requiring robust structural support; potential for rapid degradation, limiting its lifespan in specific tissues; requires careful monitoring during polymerization to avoid adverse effects	[104]
	Recombinant elastin repeat proteins (HE5c and HRGD ^d^)	Functional components incorporated into biomedical materials to promote specific functions during tissue regeneration	Myocardial infarction	Advantages: HE refers to a protease cleavage site that allows controlled degradation of the material by specific enzymes, thereby facilitating tissue remodeling in response to local enzymatic activity.	The presence of cleavage sites can generate fragments that, if not completely biocompatible, could induce unwanted immune or inflammatory responses.	[105]
IEDDA ^e^	Chitosan	Cell fixation	Skin defects	Advantages: Faster and higher-quality healing.	Chitosan requires specific pH conditions to remain soluble; for example, it is soluble in acid but insoluble under neutral physiological conditions, which may limit its direct use in specific biomedical applications. Although generally biocompatible, some modifications or conditions may induce adverse reactions.	[106]
Thiol-ene	Sodium alginate Polyethylene glycol	Sequential release	Skin defects	Advantages: Reduced volume of hypertrophic scar tissue.	Although sodium alginate is biocompatible, the chemical modifications required for it to participate in bioorthogonal reactions can alter its biocompatibility or generate toxic waste if not adequately controlled.	[107]
	Polyhydroxyal-canoates. Polyethylene glycol diacrylate	Amphipathicity, resistance to fatigue	Vascular graft	Advantages: Long-term intravascular permeability. They can be modified to exhibit contraction and expansion properties in response to changes in the aqueous or oily environment, allowing for control of their volume and shape.	The structure of polyhydroxyalkanoates is not biodegradable under physiological conditions without further modification, which may limit applications that require absorption of the biomaterial by the body.	[108]

^a^ Dibenzocyclooctyne; ^b^ Bicyclo [6.1.0]nonyne; ^c^ Protease cleavage site; ^d^ Functional domains for cell adhesion; ^e^ Inverse electron-demand Diels–Alder.

In this regard, it is clear that the viability of hydrogels depends not only on the chemical nature of their polymer networks but also on the residual byproducts after synthesis [109]. For example, in photochemically initiated systems (such as the thiol–ene mechanism) that use Irgacure initiators (chemical compounds employed for photopolymerization), radicals may be released that generate oxidative stress and cytotoxic aromatic compounds, affecting cell viability; moreover, residual thiol groups can react with endogenous proteins, thereby altering redox homeostasis and triggering inflammatory responses [110,111].

On the other hand, evaluating the immune response to implanted hydrogels is critical to the clinical success of the material. That is, the stiffness of the material and the degree of crosslinking are crucial factors influencing macrophage polarization: highly crosslinked matrices tend to induce pro-inflammatory M1 phenotypes, whereas less crosslinked matrices promote the transition toward M2, which is associated with cellular regeneration [112,113]. Additionally, the surface chemical characteristics of hydrogels (including the presence of charged triazole rings) can generate molecular patterns unrecognized by the organism, triggering lymphocyte activation [114,115].

#### 3.1.1. Relevance of Hydrogels Based on SPAAC-Type Reactions in Regenerative Medicine

The development of the SPAAC mechanism has been fundamental to the advancement of bioorthogonal chemistry, and, with the contributions of Bertozzi et al., it has enabled various modifications of biomaterials and the synthesis of potential biomaterials for medical applications [116]. Initially, the synthesis of cycloalkynes involved the use of 1,1-dibromo to introduce substituents at positions adjacent to the alkyne triple bond and thereby increase reactivity. Still, this approach was inadequate for achieving such specific substitutions on the substrate [117]. These types of substitutions are fundamental for the development of feasible, efficient, and scalable methodologies.

**Figure 4:**
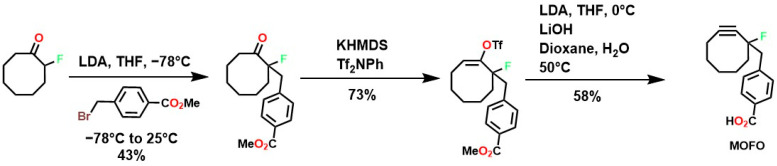
Synthesis of MOFOs by vinyl triflate substitution reactions.

To overcome these limitations, fluorine was used to achieve highly specific substitutions and improve electronic and structural characteristics. This was accomplished through reactions with vinyl triflates such as 3,3-dimethyl-1-buten-2-yl triflate, which, in the presence of pyridine, yields tert-butylacetylene [118]. Methodologies such as vinyl/enol triflate elimination have resulted in several complex structures. For example, Bertozzi et al. employed this methodology using potassium bis(trimethylsilyl)amide (KHMDS) substituted cyclooctanones and a triflate source, as shown in Figure 4.

**Figure 5:**
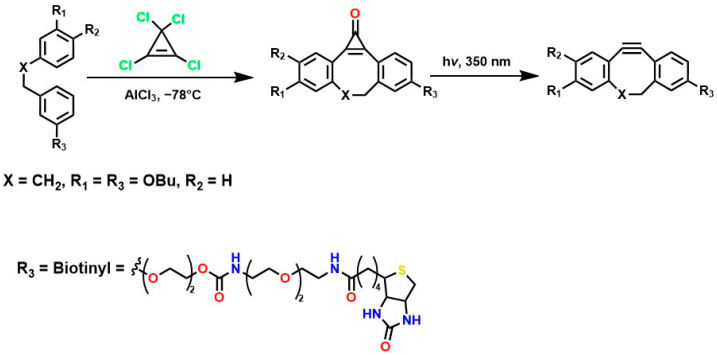
Synthesis of DIBOs by photodecarboxylation reactions.

The incorporation of a fluorine atom into cyclooctynes (predicted MOFO LD_50_: 1000 mg/kg) improves the electronic properties and reactivity of the compound; however, high yields (>80%) are required for surface and/or structural modifications to avoid significant cytotoxic effects caused by residual MOFO [119]. Furthermore, photodecarboxylation reactions of cycloprofenones have been reported to synthesize dibenzocyclooctin derivatives known as DIBOs (Figure 5) [120]. These compounds are synthesized via Friedel-Crafts reactions between 3,3′-bisbutoxybibenzyl and tetrachlorocyclopropene in the presence of AlCl_3_, followed by acid hydrolysis, yielding 23% [121,122]. These compounds exhibit biocompatible properties and have been used for cell labeling via photocatalyzed click reactions, enabling the synthesis of a variety of DIBOs via radiation-induced decarboxylation at 350 nm [123].

**Figure 6:**
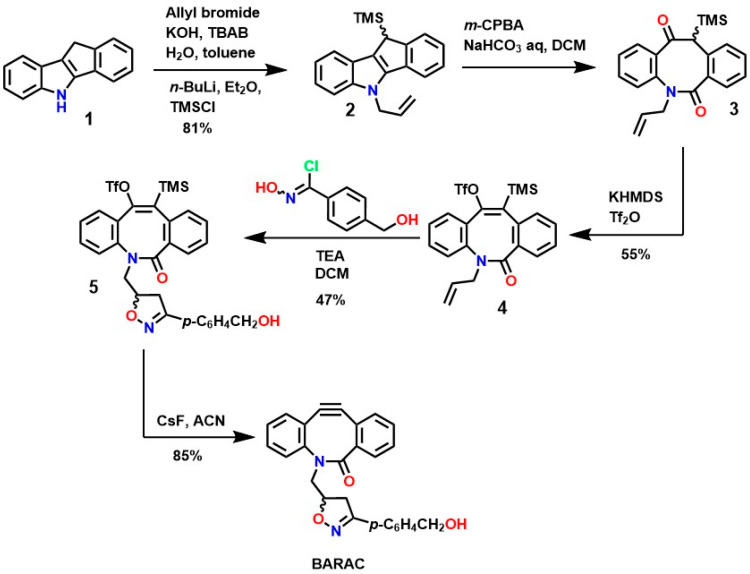
Synthesis of biarylazacoctinone (BARAC) via the triflates-enol elimination mechanism.

Similarly, several protocols have been established for the synthesis of new cyclooctynes such as biarylazacoctinone (BARAC), as shown in Figure 6. The synthesis of this compound was carried out starting from 4-dibenzocyclooctinol (DIBO) [120]. This type of compound exhibits greater reactivity due to an increase in ring deformation energy, as a greater degree of unsaturation is introduced, which can also facilitate further functionalizations [124,125].

Indole **1** (5,10-dihydroindene [1,2-b]indole), although commercially available, has an LD_50_ of 229 mg/Kg and a yield of 81%, making it ideal for the synthesis of various BARAC compounds via Fischer indole reactions. Furthermore, N-alkylation is carried out using allyl bromide and tributylammonium bromide (TBAB), and the trimethylsilyl group is coupled using trimethylsilicol (TMSCl), followed by deprotonation using n-BuLi, resulting in compound **2**. Oxidation of this compound using metachloroperbenzoic acid (m-CPBA) leads to ring opening, resulting in the formation of ketoamides **3**, and subsequent enolate formation using KHMDS, followed by treatment with Tf2O, yielding an intermediate enol triflate **4** [126]. Subsequently, treatment with triethylamine (TEA) and chlorooxime yields intermediate **5**, which exhibits the desired structural characteristics for various functionalizations. The formation of the triple bond of the compound BARAC (with an 85% yield) is achieved using CsF and a moderate reaction time, making BARAC ideal for various modifications or bioconjugation reactions [127]. However, although this process generates compounds of interest in medicinal chemistry, it requires several steps with variable yields, significantly increasing synthesis costs and imposing a prohibitive condition for industrial scalability.

**Figure 7:**
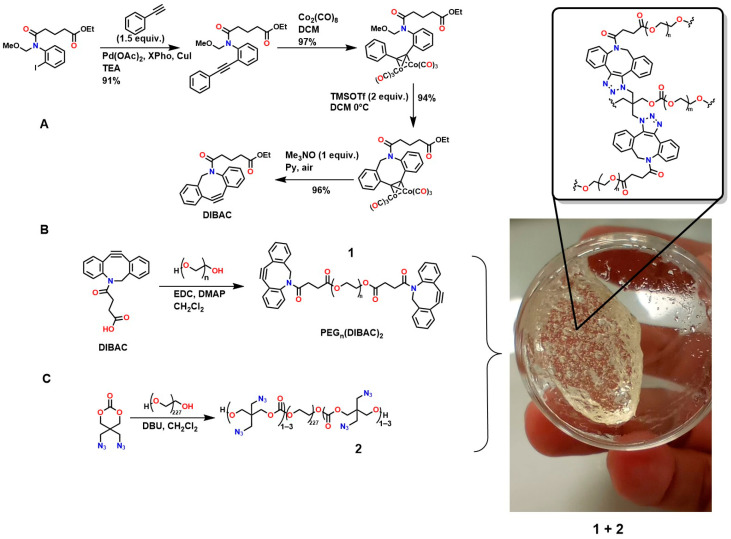
Synthesis of biocompatible hydrogels using biomedically relevant SPAAC reactions. (**A**) Synthesis of dibenzoazcyclooctyne (DI-BAC) via complex decomplexation reactions; (**B**) Modification of DIBAC with polyethylene glycol (PEG); (**C**) Polymer structure suitable for hydrogel formation via the cyclooctyne triple bond.

Furthermore, Figure 7A shows the synthesis of dibenzoazcyclooctyne (DI-BAC) via complex decomplexation reactions, which exploits the ring strain of sp^2^-hybridized atoms of the benzene rings fused to the cyclooctyne, thereby increasing its reactivity [128]. Additionally, this structure contains an endocyclic nitrogen atom that enhances its hydrophilicity. This polymer is suitable for PEGylation reactions, which are appropriate for the surface modification of various biopolymers [129,130].

Figure 7B shows the modification of DIBAC with polyethylene glycol (PEG), resulting in the compound PEG_n_(DIBAC)_2_, which has a polymeric structure suitable for hydrogel formation by utilizing the cyclooctyne triple bond, as shown in Figure 7C. The synthesis of this compound occurs through the reaction between 5,5-bis(azidomethyl)-1,3-dioxan-2-one and PEG_10K_(N_3_)m with m = 6, 9, 12. These hydrogels exhibit rapid gelation rates (within a few minutes), making them attractive for application as injectable hydrogels [59,131]. The cell viability of these polymers was studied by Hodgson et al., who performed in vitro assays using the tetrazolium dye triazolium blue tetrazolium bromide (MTT), demonstrating low cytotoxicity in 3T3 mouse fibroblasts [132]. However, the methodologies used to design and synthesize these polymers require several steps that demand strict conditions, highly expensive reagents, and various characterization and purification techniques, thereby hindering their industrial scalability for biomedical applications.

#### 3.1.2. Relevance of Hydrogels Based on Thiol–Ene/Thiol–Yne Reactions in Regenerative Medicine

**Figure 8:**
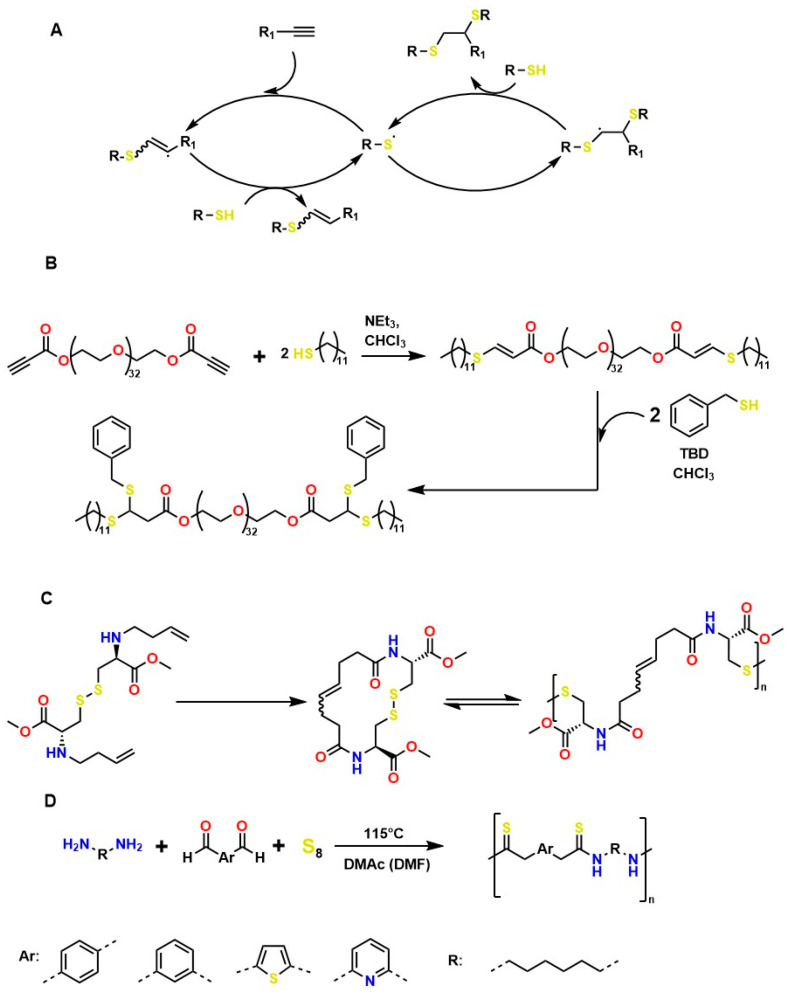
Mechanisms of Thiol–Ene and Thiol–Yne Reactions in Material Synthesis: (**A**) General mechanism of terminal alkyne dithioether reactions under radical conditions. (**B**) Sequential addition of thiols to PEG-bispropiolate. (**C**) Disulfide metathesis polymerization driven for the synthesis of high-molecular-weight heterofunctional poly(disulfide). (**D**) Diamines used in Willgerodt–Kindler-type reactions of dialdehydes in the presence of sulfur.

Several authors have reported the synthesis of sophisticated molecular structures via click-type reactions that avoid the use of Cu^+^, in which thiol and alkene groups act as initiators in a thiol–ene click reaction. This method provides suitable conditions for the formation of orthogonal networks due to its high efficiency, fast reaction rates, strong selectivity, absence of a required initiator, resistance to oxygen and moisture, and superior biocompatibility. For example, Mutlu et al. extensively addressed sulfur chemistry in the context of materials science, leading to significant advances in the development of new polymers through concerted reactions, including thiol–ene, thiol–yne, thiol–Michael addition, disulfide crosslinking, and thiol–disulfide exchange, among others [133]. Figure 8A shows the click-type reaction mechanism for polymerization between terminal alkyne dithioethers under radical conditions.

Click reactions for thiol–yne polymerization can be classified as free-radical, amine-mediated, and transition-metal-catalyzed processes; clearly, free-radical processes are attractive for the development of biocompatible materials [134]. Depending on the initiator, the free-radical reaction can be classified as photoinitiated or thermally initiated. The mechanism of thiol–yne polymerization is similar to that of thiol–ene reactions: the addition of a thiyl radical to an alkyne group is followed by hydrogen abstraction from another thiol group by carbon-centered radical species generated as a result of the initial addition [61].

It is worth noting that thiol–yne addition reactions initiated by free radicals are affected by adverse reaction conditions, low selectivity, and the formation of byproducts; bases (and nucleophiles) are the most efficient catalysts, with minimal propensity for side reactions. For example, Truong et al. describe the Michael (nucleophilic) addition of thiol–yne, analogous to the radical-mediated thiol–yne reaction, as shown in Figure 8B, where starting from dipropiolate of oxybis(ethane-2,1-diyl), it is possible to obtain oxybis(ethane-2,1-diyl) bis(3-(benzylthio)-3-(ethylthio)propanoate), which exhibits tunable mechanical properties, excellent for supporting various cellular loads in a wide range of physiological systems [134]. Disulfide metathesis polymerization is an unconventional technique for preparing polydisulfides, driven by entropy and metal-free, as shown in Figure 8C, which starts from methyl N-(but-3-en-1-yl)-S-(((R)-2-(but-3-en-1-ylamino)-3-methoxy-3-oxopropyl)thio)-D-cysteinate to form methyl N-((E)-8-(((R)-1-methoxy-3-(methylthio)-1-oxopropan-2-yl)amino)-8-oxooct-4-enoyl)-S-methyl-L-cysteinate, a self-adjusting polymer with tunable mechanical properties and heteroatoms that help improve its biocompatible properties. This disulfide metathesis polymerization is highly valued in materials science because it is remarkably fast, achieving monomer conversion rates of 70–90% within 1 to 5 min [135].

The synthesis of high molar mass heterofunctional polydisulfides, specifically poly(ester-disulfide-alkene) and poly(amide-disulfide-alkene), has been reported with values between 44,000 and 60,000 g/mol and a polydispersity index (PDI) greater than 1.7. These polymers, characterized by their thiol ending, exhibit excellent structural stability; however, the materials in Figure 8A undergo rapid depolymerization in the presence of mildly polar solvents (and in physiological media); in addition, they may exhibit oxygen inhibition, photoinitiator interference, and a strong odor from handling thiols, among others. The materials in Figure 8B (activated thiol-ene Michael reactions) generate residual acrylates upon degradation, which can alkylate proteins. The disulfide intermediates in Figure 8C (metathesis/redox) undergo structural weakening in reducing media. All these conditions limit their in vivo applicability for tissue regeneration [135]. However, the Willgerodt-Kindler reaction (Figure 8D) enables the introduction of thiocarbonyl groups (-C(=S)-NH-) into organic molecules, thereby generating functionalized surfaces for biomolecular immobilization and providing chemical flexibility to adjust mechanical and biochemical properties [136,137,138]. These transformations are notable for their high yields and absence of toxic byproducts (which favors biocompatibility), but they require high-temperature operating conditions, which limit their medical applications.

For example, Brown et al. reported on the synthesis of photopolymerized hydrogels with adjustable viscoelastic properties through thioester exchange reactions. For this purpose, polyethylene glycol diacrylate (PEGDA) was used in combination with dithiothreitol, yielding a hydrogel based on thiol-ene click reactions with glucose-responsive and self-healing properties [139]. This hydrogel was successfully applied in vivo in brain tissue engineering, where endothelial and neural stem cells produced vascularized neural tissue after 14 days of implantation [140]. However, thiol-ene and thiol-yne reactions, which are generally radical mechanisms, are susceptible to oxygen inhibition. Furthermore, in photoinitiated systems, some initiators can exhibit cytotoxicity if not strictly controlled, and excess thiols can cause unpleasant odors and handling problems, limiting applications in soft tissues [133].

Recently, thiol-ene click reactions have been used to fabricate cell matrices based on poly(oligoethylene glycol methacrylate), incorporating adhesive peptides RGD and REDV, as well as alkene-linked residues (allyl and norbornene). This approach has been highly valued for the controlled configuration of biomolecules within the polymer, which enables modulation of cell adhesion. The resulting hydrogels showed good affinity for human umbilical vein endothelial cells, exhibiting greater adhesion to structured polymer surfaces with RGD patterns [141]. Other notable examples include poly(propylene fumarate) (PPF)-based hydrogels grafted onto keratin via thiol-ene click reactions, which enabled the synthesis of chitin-based hydrogels that successfully incorporated bioactive molecules such as parathyroid hormone, functioning as vehicles for controlled and localized release, However, their applications are currently limited to in vitro studies [142].

#### 3.1.3. Relevance of Diels–Alder (DA) Reaction-Based Hydrogels in Regenerative Medicine

**Figure 9:**
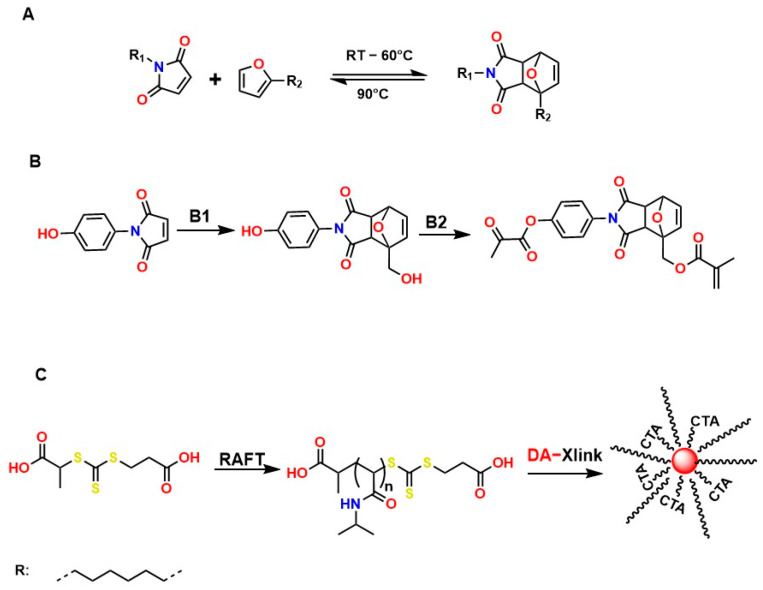
Diels–Alder-type reactions: (**A**) Reversible Diels–Alder adduct whose formation depends on temperature. (**B**) Synthetic route for the preparation of DA-Xlink in furfuryl alcohol, ACN, 45 °C, 24 h. CTA: 2-(2-carboxyethylsulfanylthiocarbonylsulfanyl) propionic acid and (**C**) polymerization of N-isopropylacrylamide (NIPAM) through a reversible fragmentation-addition chain transfer (RAFT) polymerization mechanism and is added to compound DA (DA-Xlink).

Diels–Alder (DA)-type reactions are also highly valued for the development of materials with potential applications in regenerative medicine [143,144,145]. In the DA reaction mechanism, a dienophile and a diene undergo a 4 + 2 cycloaddition to form a six-membered ring. The electronic configurations of the diene and the dienophile determine the thermodynamic parameters for both the formation and cleavage of the ring structure. Figure 9A illustrates one of the most common examples involving furan and maleimide in furfuryl alcohol, ACN, at 45 °C for 24 h, to generate the compound 4-(4-((Methacryloyloxy)methyl)-1,3-dioxo-1,3,3a,4,7,7a-hexahydro-2H-4,7-epoxyisoindol-2-yl)phenyl Methacrylate, as shown in Figure 9B. Figure 9C shows that N-isopropylacrylamide (NIPAM) is polymerized through a reversible addition–fragmentation chain transfer (RAFT) polymerization mechanism and added to the DA compound (DA-Xlink) with a certain conversion percentage during polymerization, to link the linear PNIPAM chains into nanogels and star-like structures [146,147,148,149].

**Figure 10:**
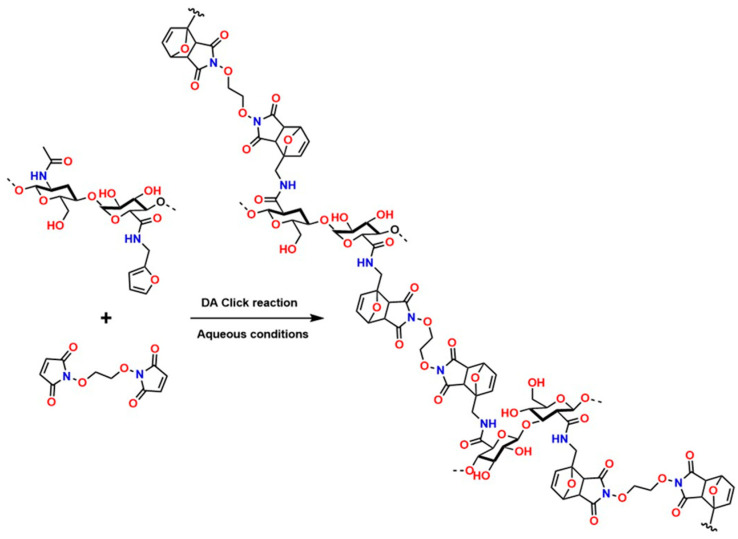
Chemical crosslinking of hyaluronic acid with poly (ethylene glycol) dimaleimide via DA-type click reaction.

Recently, several studies have highlighted the efficiency of hydrogels crosslinked through DA-type reactions for applications in soft tissue regeneration. For example, Ruiz-Pardo et al. designed a crosslinking system for polymers with polysaccharide structures, such as chitosan, under aqueous conditions that is simpler and more efficient, allowing the replacement of glutaraldehyde and avoiding its adverse effects in physiological systems, thereby generating the highly crosslinked porous structures characteristic of hydrogels. The authors produced hydrogels from hyaluronic acid via DA-type click reactions [150,151]. The hydrogels obtained from hyaluronic acid modified with furan were synthesized and crosslinked using poly (ethylene glycol) dimaleimide, as shown in Figure 10.

## 4. Chitosan Hydrogels Based on Click Reactions: Design, Functionality, and Applications in Regenerative Medicine

**Figure 11:**
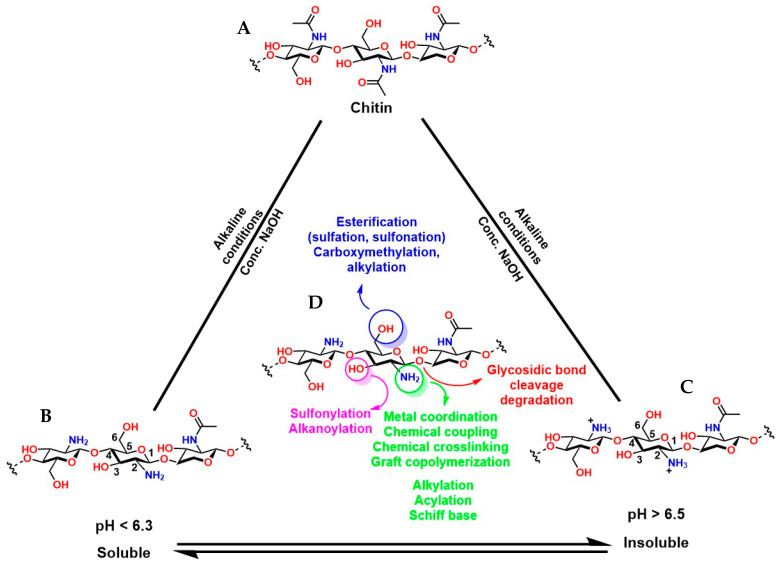
Chemical structure of chitin (**A**), its deacetylated forms (**B**,**C**), and the functionalized groups of its deacetylated form that can be chemically modified (**D**).

Chitosan (known as a copolymer composed of N-acetylglucosamine (2-(acetylamino) −2-deoxy-d-glucopyranose) and glucosamine (2-deoxy-2-amino-d-glucopyranose) units), obtained by the partial deacetylation of chitin (Figure 11A), is the second most abundant biopolymer after cellulose. The presence of N-acetylglucosamine and glucosamine units in its structure provides it with reactive functional groups: -NH_2_ at the C-2 position of the glucosamine unit and -OH groups at the C-3 and C-6 positions. This functional arrangement confers excellent chemical and biological versatility, which is key for medical applications (Figure 11B,C). The -NH_2_ groups can react with anhydrides or acid chlorides to form amides [152], with aldehydes to form imines [153], with alkyl halides to form quaternary ammonium salts [154], with isocyanates to form ureas, among others [155]. The -OH groups can react with acids or anhydrides to form esters [156], with alkyl halides or epoxides to form ethers [157]. These chemical reactions are key to synthesizing functionalized derivatives with specific properties for medical applications. Furthermore, condensation or crosslinking reactions of agents such as glutaraldehyde and epichlorohydrin allow the formation of three-dimensional structures that mimic the extracellular matrix and are fundamental in tissue engineering [158,159].

It is essential to highlight that the degree of deacetylation (DD) of chitosan is a key factor in the functionality of hydrogels (porous three-dimensional structures usually generated from condensation reactions) prepared from this polymer. A high DD implies a greater number of available -NH_2_ groups, which not only increases its chemical reactivity towards click-type reactions [160], but also enhances protonation under acidic conditions, generating a greater positive charge in the material that promotes cell adhesion [161]. pH control is fundamental because under strong pH conditions (very acidic), the protonation of the amine groups (-NH_3_^+^) increases the viscosity of the polymer, while at alkaline pH, protonation and electrostatic repulsions are reduced, favoring pseudoplastic properties in the material, which is attractive for the fabrication of scaffolds in tissue engineering and 3D bioprinting [162]. Furthermore, it is possible to adjust structural characteristics and modulus of elasticity [163], swelling and estrogenic sensitivity [164], gelation kinetics [165], compatibility, and cell proliferation [161]. However, complete pH control is essential because a high electrostatic charge in the material can lead to nonspecific interactions with plasma proteins in physiological environments, as well as accelerate degradation catalyzed by lysozymes and chitinases [161,166,167,168,169,170]. On the other hand, chitosan with a lower DD (i.e., more acetylated) is less susceptible to enzymatic hydrolysis, thus extending the hydrogel’s shelf life. Conversely, higher values favor faster degradation, which can be advantageous in temporary applications such as matrices for controlled release [171].

Several studies have demonstrated that intrinsic characteristics of chitosan, such as molecular weight, viscosity, concentration, and the solvents used to dissolve the polymer (such as acetic acid, citric acid, and/or buffers like AcOH-NaAc), as well as the ionic strength of the medium, exert a direct effect on the antimicrobial properties of chitosan [172,173]. These factors modify solubility, surface charge, and interaction with cell membranes, which determine the material’s efficiency. The length of the polymer chain also plays a crucial role in this activity: Chitosan oligosaccharides with molecular weights of approximately 2200 Da have been reported to stimulate the growth of E. coli, while oligosaccharides with molecular weights of approximately 9300 Da exhibit significant antimicrobial properties [174]. This behavior is explained by the ability of longer chains to generate a higher charge density, favoring electrostatic interactions with cell membranes [162]. In physiological media, it is essential to highlight chitosases (EC 3.2.1.132) and chitinases (EC 3.2.1.14), responsible for the endohydrolysis of the β-(1→4) linkages of D-glucosamine in partially acetylated chitosan [175,176]. These enzymes degrade the polymer, preventing accumulation and fibrous responses, which is crucial because the degradation rate of the material must be synchronized with the generation of new tissue and the formation of extracellular matrix, ensuring appropriate conditions for tissue engineering applications [163].

Among the most common modifications of chitosan are N-substitutions and O-substitutions, which allow for the adjustment of characteristics such as solubility, charge density, and functionality for biomedical applications [177]. A particularly noteworthy N-substitution reaction occurs with N,N,N-trimethylchitosan chloride (TMC), which yields one of the most relevant quaternary chitosan derivatives due to its excellent solubility in aqueous media and its functional versatility in biomedical applications [178]. This polymer not only acts as an adsorption enhancer in controlled-release systems but also exhibits antibacterial properties and the ability to contain and transport genetic material, making it a strategic candidate for advanced therapies as a non-viral vector for gene delivery and as a platform for tissue engineering [179].

The synthesis of TMC can be approached through different methods to optimize quaternization and minimize side reactions that could compromise the material’s functionality. Direct one-step methylation involves the reaction between chitosan and methyl iodide (CH_3_I) under strongly alkaline conditions using N-methyl-2-pyrrolidone (NMP) as a solvent. Although this reaction is operationally simple, it carries the risk of inducing O-methylation reactions that can alter the solubility and bioactivity of the material [180].

**Figure 12:**
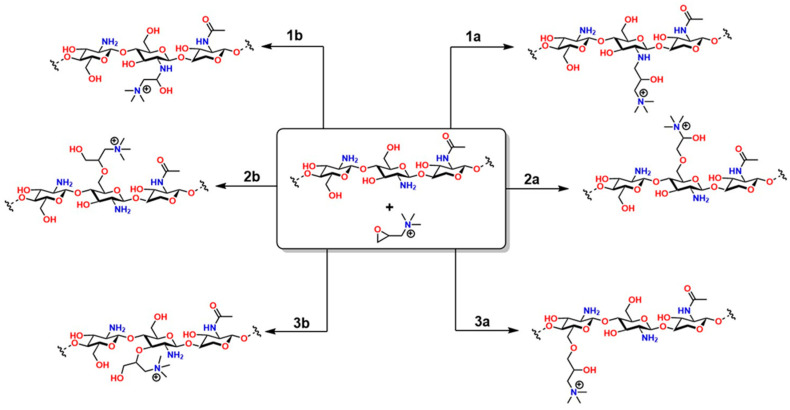
Types of reactions between chitosan and Eptac. All products obtained in this reaction are attributed to chitosan’s functional groups. Adapted from Yang et al. [184].

The two-step method offers greater regioselective control: first, N-methylchitosan or N,N-dimethylchitosan is generated by forming a Schiff base with formaldehyde in an acidic medium, followed by a methylation reaction with CH_3_I [181]. The choice of method depends on balancing synthetic simplicity and structural control, critical factors for ensuring the material’s effectiveness in pharmaceutical and nanotechnology applications [182]. On the other hand, the reaction between chitosan and 2,3-epoxypropyl trimethyltrimethyl (Eptac), catalyzed by 1-allyl-3-methylimidazolium, allows for several addition reactions on chitosan (Figure 12): in steps 1a and 1b, Eptac reacts with the NH_2_ group of chitosan, introducing a quaternary ammonium group (-N^+^(CH_3_)_3_), converting the primary amines into quaternary ammonium salts, thus improving the solubility of the material in aqueous media [183].

In 2a and 2b, etherification reactions occur at the -OH groups, as well as in **3a** and **3b**. These chemical modifications confer attractive biological and physicochemical properties to chitosan for medical applications, such as improved solubility, bactericidal properties, and increased chelation capacity for various metals [184]. In **2a** and **2b**, etherification reactions occur on the -OH groups, as well as in **3a** and **3b**. These chemical modifications confer attractive biological and physicochemical properties to chitosan for medical applications, such as improved solubility, bactericidal properties, and enhanced chelation capacity for various metals [184].

### 4.1. Classical Reactions in the Synthesis of Chitosan-Based Hydrogels

Hydrogels are characterized by their three-dimensional, polymer-mesh-like cross-linked structures, which create a highly porous scaffolding that allows for the diffusion of molecules and an extraordinary capacity to retain water due to the presence of branched groups such as SO_3_H, OH, NH_2_, COOH, CONH_2_, etc. [185]. The in vivo application of hydrogels began in the mid-1960s with the synthesis of (2-hydroxyethyl methacrylate) (PHEMA), which was used as a contact lens due to its moisture-absorbing capacity and excellent mechanical properties [186]. Subsequently, several authors reported the synthesis of carboxymethylcellulose (CMC)-polyvinyl alcohol (PVA)-based films for the controlled delivery of water-soluble drugs under physiological conditions, using citric acid (CA) as a low-cost, non-toxic crosslinking agent [187].

**Figure 13:**
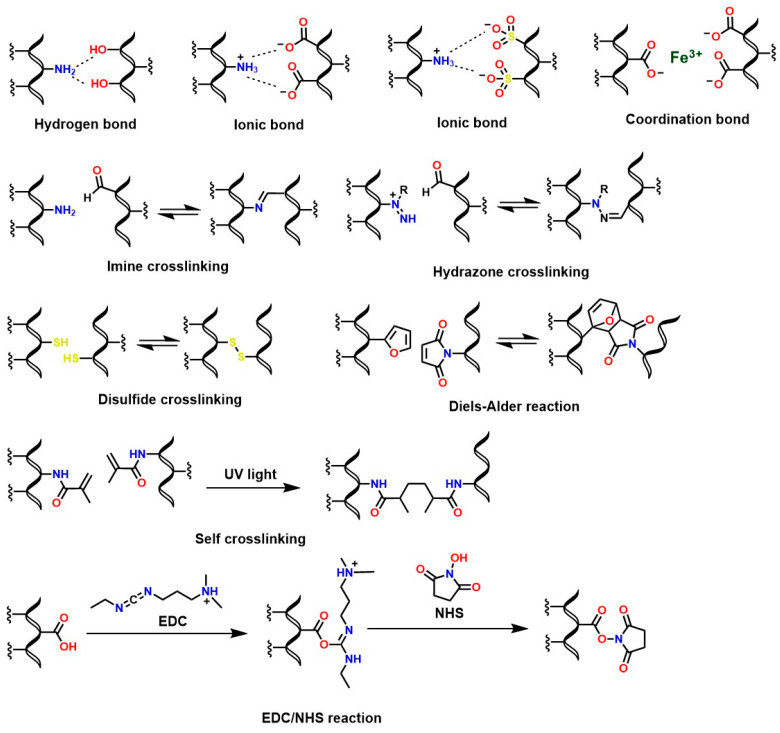
Formation of crosslinked chitosan hydrogels via classical reactions.

The polysaccharide structure of chitosan has been widely valued in the health field due to unique properties such as biocompatibility [188], osteoconductive properties [189,190], good degradation kinetics in vivo/in vitro [191], hemostatic activity [192], anti-inflammatory properties [193], antioxidant properties [194], mucoadhesive properties [195], among others. However, its low solubility at neutral and basic pH limits the fabrication of chitosan-based biomaterials for medical applications. Therefore, functionalizing chitosan is necessary to confer desirable properties to the derived materials. For example, in vivo solubility and degradability are critical aspects for the synthesis of chitosan hydrogels [196]. For this reason, several reactions have been reported to crosslink chitosan and generate hydrogels with adjustable properties based on molecular interactions such as covalent (Schiff base condensation reactions, disulfide bond formation, amidation reaction with 1-ethyl-3-(3-dimethylaminopropyl) carbodiimide (EDC)/N-hydroxysuccinimide (NHS) activation, and Diels–Alder addition reactions) and non-covalent interactions (such as electrostatic interactions and hydrogen bonds) [25,197], as shown in Figure 13.

In this context, several hydrogels with specific properties have been reported. For example, Chitosan-poly(glutamic acid)-alginate exhibits high biocompatibility and biodegradability, as well as good water retention capacity, which is appropriate because it allows for tissue hydration during implantation; however, it has limited mechanical properties, which restricts its application to tissues subjected to tension or mechanical stress [198], freeze-dried chitosan-alginate has a highly porous structure that is ideal for promoting cell migration and angiogenesis; however, it exhibits mechanical fragility when hydrated [199], chitosan-κ-carrageenan exhibits rapid gelation and water retention, making it suitable for the development of injectable hydrogels in vivo. However, Chitosan-κ-carrageenan has poor mechanical properties and can generate inflammatory responses in some tissues [20,200]; chitosan-silk peptide exhibits excellent biocompatibility and cell adhesion, but it has high costs and slow degradation kinetics, which can interfere with the regeneration of injured tissues [201]; chitosan-gelatin exhibits a high similarity to extracellular matrices, which promotes cell adhesion and proliferation, but it is prone to rapid degradation in aqueous or physiological media [202]; thiolated chitosan-hyaluronic acid has mucoadhesive properties (ideal for oral and dermal applications) but the presence of thiol groups can generate cytotoxicity [203]; and diethylaminoethyl chitosan-hyaluronic acid exhibits a high surface charge that favors interaction with proteins. Still, the excess charge can develop inflammatory responses, and its gelation is complex [97,204].

Chitosan-casein phosphopeptides promote tissue regeneration, have good biocompatibility, but exhibit poor mechanical properties and possible immunogenicity effects from milk proteins [205]. Other notable examples include hydrogels such as chitosan-casein [206], chitosan-dextran sulfate [207], chitosan-chondroitin sulfate [208], chitosan-pectin [209], and trimethyl chitosan-HA-dextran sulfate-alginate [210]. However, they all present conditions that may be prohibitive for in vivo applications.

### 4.2. Chemical Modifications of Chitosan to Improve Its Properties and Overcome Limitations In Vivo Applications

**Figure 14:**
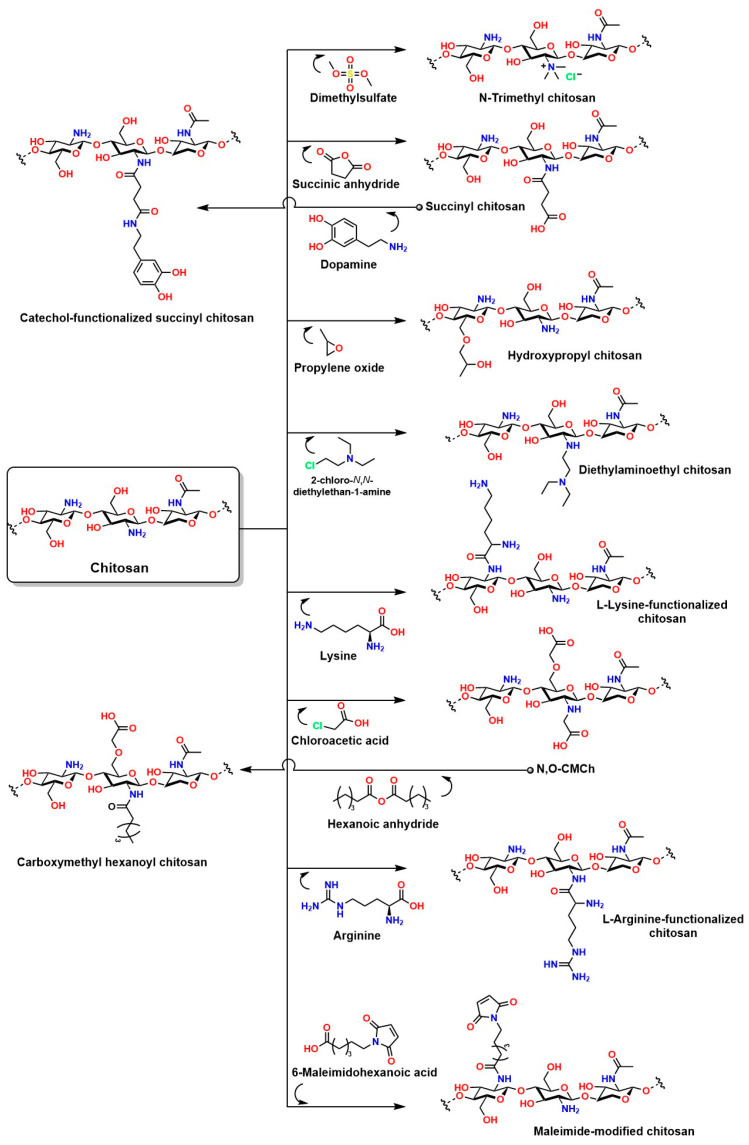
Chemical modifications to chitosan that help improve its limitations in in vivo applications.

Chemical modifications to chitosan are essential to overcome the inherent limitations of this material in in vivo applications. The presence of reactive groups such as NH_2_ and OH in the chitosan structure enables a wide variety of concerted reactions [211]. For example, L-chitosan grafted with lactic acid improves the mechanical, thermal, and water absorption properties of hydrogels compared to hydrogels prepared with traditional chitosan [212]. For applications under highly sensitive conditions requiring high material solubility (such as injectable hydrogels), chitosan grafted with amylose is an attractive option [213]. Chitosan grafted with gallic acid is ideal for enhancing cell adhesion, antibacterial capacity, and near-infrared photothermal properties, making it suitable for photothermal and photodynamic therapies [214]. Chitosan grafted with methacrylate is ideal for the development of injectable hydrogels because it can be UV-crosslinked, allowing for in vivo application during surgical procedures with adjustable crosslinking [215,216,217]. Similarly, phosphorylated chitosan can attract signaling biomolecules and undergo ion exchange; in addition, a phosphorylated chitosan coating on prostheses has been shown to improve the immune response by preventing inflammation and promoting the spread and proliferation of MC3T3-E1 cells [205]. Figure 14 shows other relevant reactions to chitosan that will enhance its properties for in vivo applications.

#### Synthesis of O-Alkyl Chitosan Hydrogels and Chemoselective Conjugations via Click-Type Reactions

Alkylation reactions on chitosan have attracted significant interest in various medical fields, primarily due to their high chemical stability and biocompatibility [218]. These modifications allow the incorporation of specific functional groups into the chitosan structure, as well as improving physicochemical properties to act as carriers for the transport and release of peptides and various bioactive molecules, making this material a versatile platform for applications in regenerative medicine, with potential uses as implant coatings and as scaffolds for injured tissues [219,220,221]. In this context, click chemistry offers a set of highly selective, rapid, and high-yield reactions for achieving more specific modifications without altering the chitosan backbone.

**Figure 15:**
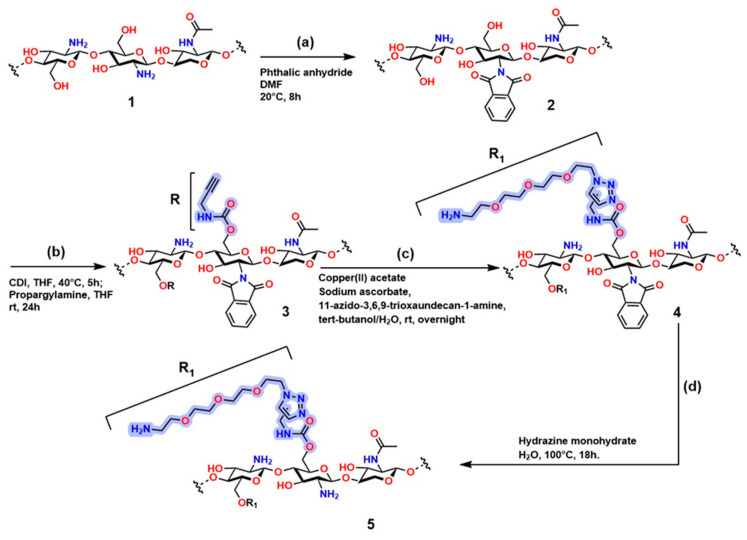
Synthetic route for chitosan modification through azide–alkyne conjugation reactions. (**a**) Protection of amino groups in chitosan; (**b**) O-alkylation of chitosan; (**c**) Concerted click reactions; (**d**) Deprotection of amino groups. Adapted from Oliveira et al. [223].

Figure 15 shows the sequence of chemical modifications of chitosan to introduce functional groups through highly selective reactions, including alkylation and click chemistry. Compound (**1**) (step a) in Figure 15 corresponds to the polysaccharide structure of chitosan; in compound (**2**), the protection of the -NH_2_ group occurs, resulting in N-phthaloyl chitosan functionalized with phthalic anhydride using the method developed by Kurita et al. [222]. Compound (**3**) (step b) shows N-phthaloyl chitosan O-prop-2-ynyl carbamate, which is obtained through a reaction with 1,1′-carbonyldiimidazole (CDI), generating a derivative containing an alkyne group (-C≡C-) that creates an anchoring point for the conditions required for click reactions. Compound (**4**) (step c) is obtained from compound (**3**) in the presence of copper acetate, sodium ascorbate, and PEG-type azide (11-azido-3,6,9-trioxaundecan-1-amine) to establish CuAAC conditions, generating the functional fragment R. Compound (**5**) is obtained from compound (**4**) using hydrazine monohydrate, producing chitosan carbamate O-(11-amino-3,6,9-undecan)triazolyl (compound **5**) [223].

The structure of this polymer is of great importance in materials science, as it contains heteroatoms that are attractive for improving its behavior in physiological systems; moreover, it exhibits an ideal porous structure for controlled and localized release of biomolecules such as growth factors and drugs. However, residual Cu in the structure limits its application in physiological systems. Therefore, chitosan modifications through O-alkylation are problematic for its use in physiological systems.

### 4.3. N-Methylated Chitosan Derivatives via Azide–Alkyne Reactions

**Figure 16:**
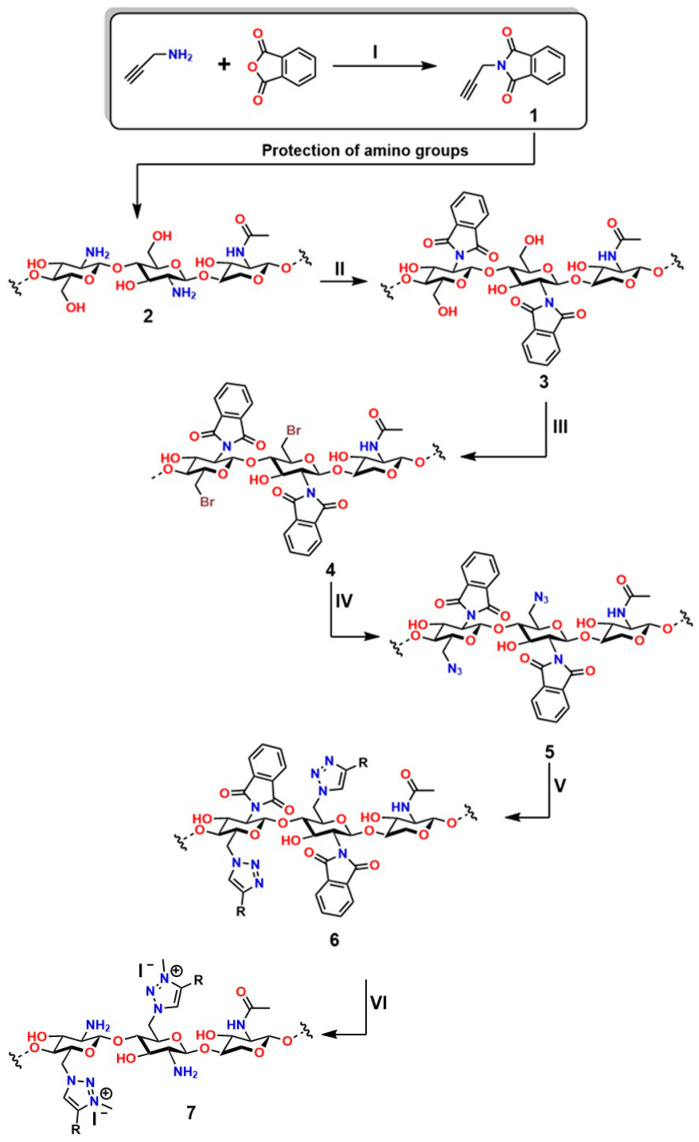
Polymer functionalization through click chemistry (Stages **I**–**VI**): synthesis of an alkyne derivative (**1**), its coupling to the polymer (**2**–**3**), bromination (**4**), substitution with azide (**5**), and formation of the triazole (**6**), characteristic of click chemistry (**6**–**7**), deprotection of -NH_2_ groups. This process illustrates how functional polymers are designed for biomedical and nanotechnological applications. Adapted from Tan et al. [234].

Chemical modifications of chitosan that exploit the reactivity of the -NH_2_ group have enabled the development of cationic materials based on 1,3,4-trisubstituted and 1,2,3-triazolium systems. These structures are obtained by alkylation of 1,2,3-triazoles with alkyl halides or alkyl triflates, yielding a broad spectrum of structural diversity with enhanced properties and expanded functionality [224,225]. Moreover, the -NH_2_ group plays a fundamental role in chitosan, as it exhibits attractive bioactive properties for applications in regenerative medicine; for this reason, preserving these groups is essential. To achieve this, phthaloyl groups are employed, which show an efficiency of 82% in dimethylformamide (DMF) with 5% (*v*/*v*) water [226], as shown in the first reaction of Figure 16.

In the first stage (**I**) of Figure 12, the synthesis of 3-phthalimidomethylacetylene (compound **1**) is shown, starting from a primary amine and phthalic anhydride, forming a structure with a terminal alkyne group and an imide ring [227]. This intermediate is used in the second stage (**II**) to react with the base polymer (**2**), which contains multiple hydroxyl and amino groups, generating N-phthaloyl chitosan (**3**) through amide linkage [228]. Subsequently, in the third stage (**III**), a bromine atom is introduced into the structure (**4**), enabling activation for nucleophilic substitution to form 6-bromo-6-deoxy-N-phthaloyl chitosan [229]. In the fourth stage (**IV**), bromine is replaced by an azide group (**5**), preparing the system for the final reaction to form 6-azido-6-deoxy-N-phthaloyl chitosan [230]. In step five (**V**), the azide-alkyne cycloaddition (CuAAC) is carried out, leading to the formation of the 1,2,3-triazole ring (**6**), characteristic of “click” chemistry. This process yields chitosan derivatives 6-(1,2,3-triazol-1-)-6-deoxy-N-phthaloyl, with high water solubility, a property particularly valued for applications in regenerative medicine [231]. Thanks to its high efficiency and selectivity under mild conditions, this strategy has become a key tool for the functionalization of biopolymers.

In step six (**VI**), the triazole compound (**7**) undergoes a further modification incorporating a functional group **R**, which enhances its capacity to amplify specific interactions and generate 6-(1,2,3-triazol-1-)-6-deoxy-chitosan derivatives [232]. In this same step, an ionic complex is formed in which the triazole ring acquires a positive charge stabilized by a counterion (I^−^). Characteristics such as these can enhance affinity for biomolecules and biodegradability, as seen in the 6-(3-methyl-1,2,3-triazolium-1-)-6-deoxy-N-phthaloylchitosan derivative. This modular approach enables fine-tuning key characteristics of these materials to ensure cell viability, transport capacity, and chemical stability. Furthermore, the residual toxicity of Cu(I) and the synthetic complexity in later stages must be considered [233]. This sequence represents a robust approach to engineering functional polymers for controlled-release and gene-delivery systems.

**Figure 17:**
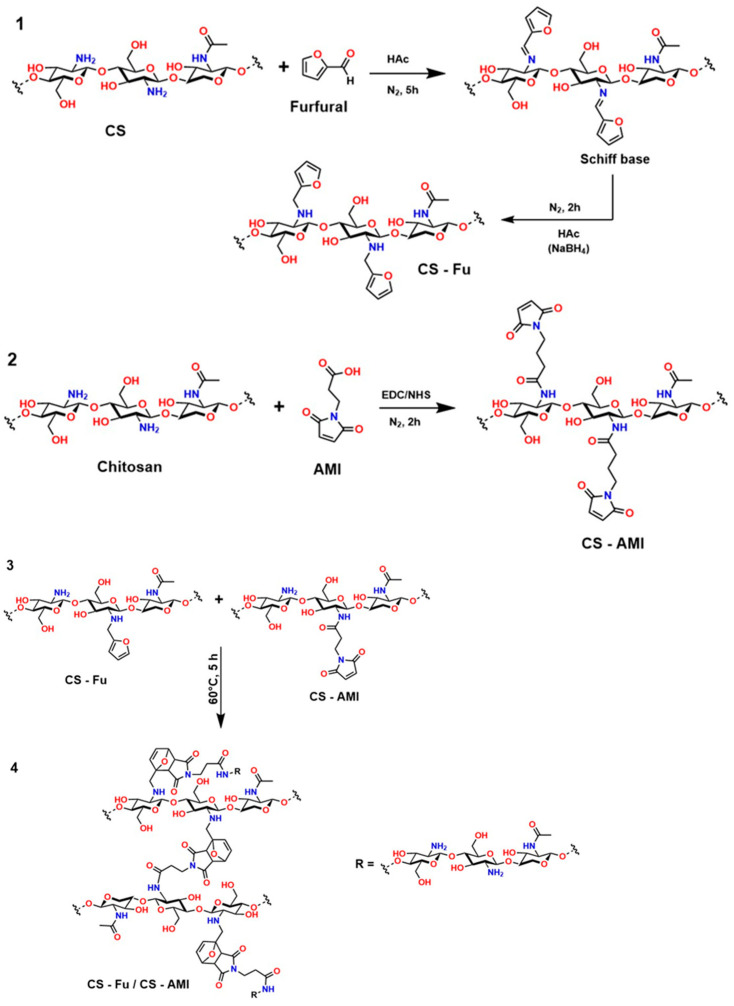
Coupling of chitosan derivatives functionalized with furfural and maleic anhydride: In (**1**), chitosan reacts with furfural in an acidic medium to generate a Schiff base, which is subsequently reduced with NaBH_4_, yielding the CS-Fu derivative. In (**2**), chitosan is functionalized with an imide derivative (AMI) through carbodiimide activation (EDC/NHS), forming the CS-AMI conjugate. In (**3**–**4**), both derivatives are combined under controlled conditions (60 °C, 5 h) to produce the hybrid material CS-Fu/CS-AMI, which features multiple functional groups (aromatic and imide) that can enhance properties such as biocompatibility, loading capacity, and structural stability. Adapted from Montiel-Herrera et al. [236].

In the first route (**1**) of Figure 17, chitosan reacts with furfural in an acidic medium (HAc) under a nitrogen atmosphere for 5 h, forming a Schiff base. This intermediate is stabilized by reduction with NaBH_4_, yielding the CS-Fu derivative. This type of modification introduces a heterocyclic aromatic group into the polymer structure, thereby enhancing properties such as hydrophobic interactions and drug retention capacity [235,236]. In the second step (**2**), 3-(2,5-dioxo-2,5-dihydro-1H-pyrrole-1-yl)propanoic acid (AMI) is grafted onto chitosan by activation with 1-ethyl-3-(3-dimethylaminopropyl)carbodiimide (EDC) and N-hydroxysuccinimide (NHS), allowing the formation of the CS-AMI conjugate [237]. This carbodiimide coupling method is widely used to link carboxyl and amino groups, generating stable amide bonds, which are fundamental in biomedical applications. In the final step of this process (**3**–**4**), both derivatives (CS-Fu and CS-AMI) react at 60 °C for 5 h, resulting in a CS-Fu/CS-AMI hybrid system [238].

This novel structure incorporates multiple functional sites, including aromatic and heterocyclic groups, which provide specific properties desirable for medical applications, such as biocompatibility, load-carrying capacity, and thermal stability. This sequential design is essential in biomaterials engineering for controlled-release systems and molecular transport. However, it is crucial to consider that Schiff base formation can be reversible if proper reduction is not performed, and that the use of agents such as EDC/NHS requires strict pH control to avoid side reactions [239].

## 5. Advances in Regenerative Medicine Based on Click Chemistry: Perspectives on Soft Tissue Healing

The use of adhesives as an alternative to traditional sutures has gained significant interest in clinical settings where minimizing surgical trauma and accelerating tissue repair are required. However, conventional adhesives present critical limitations that compromise their effectiveness, including poor tissue adhesion and insufficient mechanical strength, biocompatibility issues, and uncontrolled degradation, which affect material stability and the quality of the healing process [240,241]. These deficiencies not only reduce therapeutic effectiveness but also limit their application in complex wounds. For example, Porter et al. reported innovative methods based on bioorthogonal click chemistry to evaluate the metabolism of inflammation-affected cartilage under traumatic conditions, representing a significant advance in medical treatment [242,243].

The ideal skin adhesive must meet essential criteria: safety, rapid solidification under physiological conditions, strong tissue adhesion, and the ability to promote tissue regeneration. In this context, bioorthogonal click chemistry emerges as a promising strategy, as its high selectivity minimizes byproduct formation and enables controlled incorporation of bioactive molecules [244]. This feature opens the possibility of designing innovative adhesives that not only seal the wound but also activate key biological pathways for repair [245].

Recent studies have demonstrated the potential of SPAAC-based hydrogels to optimize wound healing. For example, Wang et al. reported significant advances in highly efficient treatments for wound healing following traumatic injuries or surgical interventions, comprising novel synthetic wound-healing peptides embedded in hydrogel dressings via crosslinking of hyaluronic acid functionalized with dibenzocyclooctyne and poly (ethylene glycol) azide. Hyaluronic acid hydrogels were grafted with these new wound-healing peptides (a functional derivative of erythroid differentiation regulator 1), which exhibited intense stimulation of cell motility, thereby sustaining the release of physiologically active peptides over a prolonged period [103]. This system activated FAK signaling, promoting the migration of fibroblasts, keratinocytes, and endothelial cells. As a result, accelerated healing and significant improvement in regenerated tissue quality were observed, as evidenced by higher epithelial cell density, increased microvascular formation, and homogeneous collagen distribution [103].

Complementarily, Shen et al. fabricated PEG thiolate/diacrylate hydrogels (BSSPD) via thiol–ene click reaction for sequential release of small extracellular vesicles (sEVs) secreted by bone marrow-derived mesenchymal stem cells (B-sEVs) [107]. In vivo studies showed that tissues treated with BSSPD hydrogels loaded with vesicles for sequential release (SR-sEVs@BSSPD) exhibited more homogeneous vascular organization, with an orderly arrangement of collagen fibers and a significant reduction in hyperplastic scar tissue volume compared with control groups and other treatments. These findings suggest that the sequential release strategy not only accelerates healing but also improves the structural quality of regenerated tissue, reducing the risk of fibrosis and promoting functional repair closer to native architecture [107].

### 5.1. Bioorthogonal Click Chemistry in Hydrogels for Nerve Injury Repair

Various injuries to the central and peripheral nervous systems trigger a series of complex pathophysiological events that hinder functional recovery. Vascular damage associated with the injury induces an inflammatory response, excessive production of free radicals, and ionic imbalance, creating a hostile microenvironment for neuronal regeneration [246]. Added to this is the formation of glial scars, resulting from reactive glial cell proliferation, which act as physical and chemical barriers, limiting axonal migration and the restoration of connectivity [247]. The low regenerative capacity of endogenous neurons necessitates advanced strategies, such as stem cell transplantation to induce neuronal differentiation or the controlled release of bioactive molecules that stimulate endogenous regeneration [248,249]. In this context, bioorthogonal click chemistry emerges as a revolutionary tool. Its high selectivity and orthogonality enable covalent bonding between cells and biomaterials without compromising cell viability or altering critical functions.

Liu et al. They developed functionalized grafts using click reactions to improve the capacity for controlled incorporation and release of drugs and biomolecules for applications such as spinal cord implants [250]. This system showed advantages compared to traditional materials, where astrocytes and neural progenitor cells (NPCs) covalently anchored to collagen fibers exhibited stronger adhesion, greater extension, and differentiation, promoting cell integration and tissue regeneration. Furthermore, the inclusion of lipid nanoparticles functionalized with DBCO groups via the SPAAC reaction allowed for the localized delivery of edaravone (a compound with antioxidant properties ideal as a protective agent in situations of oxidative stress). This approach significantly reduced the expression of pro-apoptotic proteins and lipid peroxidation markers at the regeneration site, improving cell survival. In addition, the functionalized graft promoted key processes such as neurogenesis, axonal regeneration, and blood vessel formation, which are fundamental for structural and functional recovery [251].

On the other hand, Zhang et al. designed a biodegradable self-adhesive bandage (SADB) loaded with XMU-MP-1 nanoparticles, a Hippo pathway inhibitor that promotes cell proliferation and nerve repair. Thanks to click chemistry, the bandage adhered directly to the transected nerve in a rat sciatic nerve transection model, reducing surgical time and improving procedural precision. Electrophysiological analyses and histological studies confirmed superior functional recovery, evidenced by higher nerve conduction velocity, reduced latency, and efficient myelin regeneration compared to the control group [104].

### 5.2. Bioorthogonal Click Chemistry in Hydrogels for Vascularization in Tissue Engineering

The viability and functionality of tissue grafts depend on an adequate supply of oxygen and nutrients, making vascularization a critical factor for successful tissue regeneration. Insufficient angiogenesis or limited vascular perfusion is a common cause of graft failure, especially in those designed to repair complex tissues [252]. Therefore, inducing a functional vascular network within the graft is crucial to ensure its integration with host tissue and prevent necrosis.

Several studies have demonstrated that bioorthogonal click chemistry can be used to crosslink hydrogels and anchor pro-angiogenic biomolecules without compromising biocompatibility. Jia et al. functionalized alginate hydrogels with the α1 peptide via CuAAC reaction, creating a platform that promotes adhesion, migration, and proliferation of endothelial cells (ECs). In murine models of hindlimb ischemia, this system increased vascular network formation and reduced muscle fibrosis, highlighting its potential to restore tissue perfusion [253]. Complementarily, Marsico et al. designed elastin-like hydrogels by linking two ELR polypeptides containing MMP and integrin recognition sites through SPAAC reaction. This approach not only increased capillary and arterial density in severe ischemia models but also regulated essential biological processes such as endothelial proliferation and extracellular matrix remodeling, contributing to more efficient tissue repair [254].

### 5.3. Bioorthogonal Click Chemistry in Hydrogels for Cardiac Regeneration

Stent implantation remains the standard treatment for coronary artery disease, but its use presents critical limitations, such as in-stent restenosis, late thrombosis, and incomplete endothelial healing [255,256]. These issues highlight the urgent need to develop coatings that not only prevent thrombotic complications but also promote rapid endothelial regeneration [257].

Furthermore, hydrogels based on natural polymers such as chitosan have established themselves as highly versatile platforms with promising applications in regenerative medicine. For example, chitosan is highly valued due to its bioavailability, biodegradability, and cell compatibility, which positions it as an ideal material for the synthesis of hydrogels with a wide variety/diversity of molecules due to its structure rich in -NH_2_ and OH groups that facilitates the incorporation of bioactive molecules through specific reactions, such as bioorthogonal “click” chemistry, without affecting cell viability [258,259,260,261].

The potential of catechol-modified chitosan as a base for multifunctional coatings on coronary stents. This system enabled efficient immobilization of the REDV peptide (known to promote endothelial cell adhesion and proliferation) via a thiol–ene click reaction. Additionally, the coating catalyzed the controlled release of nitric oxide (NO), a key gasotransmitter in thrombosis prevention and vascular tone regulation [262]. In animal models, this approach drastically reduced stent occlusion and maintained blood flow integrity while suppressing excessive intimal proliferation and preserving the contractile phenotype of smooth muscle cells. These findings confirm that functionalized chitosan hydrogels can act as bioactive interfaces, combining antithrombotic properties with regenerative stimuli.

In addition to their frequent use in the regeneration of coronary tissue following myocardial infarction, their hydrated three-dimensional structure facilitates the retention of growth factors, extracellular vesicles, and stem cells, creating a favorable environment for angiogenesis and tissue repair [236]. Through bioorthogonal click chemistry, these hydrogels can be modified to incorporate pro-angiogenic peptides, proteins such as VEGF, and even antithrombotic drugs like bivalirudin, ensuring controlled and prolonged release at the site of injury [263]. Furthermore, SPAAC reactions, for example, enable crosslinking of the hydrogel and immobilization of biomolecules under physiological conditions, avoiding the use of toxic metal catalysts and preserving the biological activity of the functional components. This characteristic is crucial for in vivo applications, where safety and efficacy are paramount. In preclinical studies, click chemistry-based systems have shown improvements in ventricular ejection fraction, reduced fibrosis, and increased capillary density in ischemic areas, demonstrating their positive impact on functional myocardial recovery [20].

## 6. Future Perspectives

**Figure 18:**
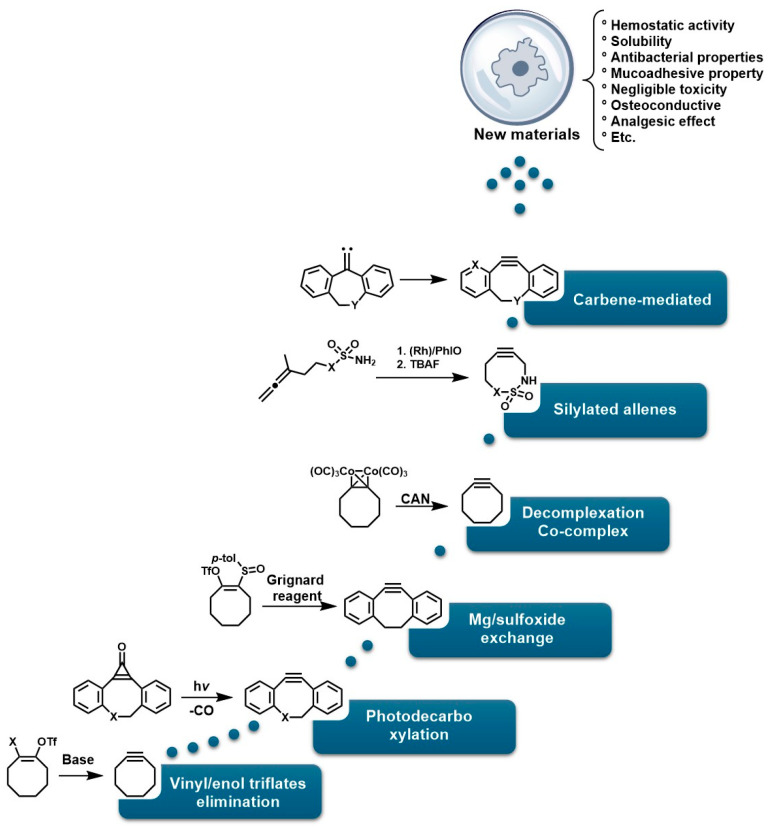
Types of click reactions relevant to the synthesis and functionalization of potential biomaterials for applications in regenerative medicine.

The development of functionalized biomaterials through click-type reactions is an emerging field currently at an advanced stage of chemical maturity; however, chemical challenges persist that remain prohibitive to their complete application in highly invasive surgical interventions or advanced biomedical applications. Future research on this topic should focus on greater convergence between molecular design and biological function to overcome current limitations in the applicability of these materials and to facilitate their in vivo applications [264]. For example, several prohibitive barriers to the industrial scalability of materials produced by this type of reaction are shown in Figure 18. The synthesis of cyclooctyne derivatives (such as MOFO, DIBOs, BARAC, DI-BAC) takes place through vinyl/enol triflates elimination, photodecarboxylation, Mg/sulfoxide exchange, decomplexation co-complex, silylated allenes, carbene-mediated reactions, among others. These reactions expand the possibilities for surface functionalization across a wide variety of biopolymers and potential biomaterials for in vivo medical applications. Still, they require a series of sequential steps and controlled conditions that considerably increase costs (500–1000 USD/g), hindering the scalability of these reactions for the synthesis or functionalization of materials [27].

In other words, for these materials to be commercially available, they must strictly comply with basic requirements and quality-control standards (GMPs). If the materials are manufactured and/or functionalized using CuAAC, strict GMP controls and quality management systems (QMS) must be implemented to ensure the complete elimination of residual copper in the materials to avoid inflammatory responses and reactive oxygen species (ROS). Furthermore, a significant problem associated with regioselectivity arises when using asymmetric alkynes, as it is difficult to control and increases production costs [57]. For example, the synthesis of precursors for click reactions requires several steps, and the reagents are expensive. A query on Sigma Aldrich (St. Louis, MO, USA) (www.sigmaaldrich.com (accessed on 30 November 2025)) shows that some precursors, such as Dibenzocyclooctyne-PEG4-maleimide (ideal for biorthogonal reactions via SPAAC), have costs of 48.61 USD/g, Alkyne-PEG4-maleimide 76.82 USD/10 mg, SM(PEG)_24_ (PEGylated, long-chain SMCC cross-linker) 770.86 USD/100 mg, DBCO-dPEG^®^_12_-MAL 356.60 USD/25 mg, precursors for Diels–Alder-type reactions: MAL-dPEG^®^_8_-acid 398.96 USD/100 mg, and for thiol/ene reactions: Thiol-dPEG^®^_12_-acid 464.87 USD/100 mg.

It is also important to highlight that optimizing polymer networks is essential to achieve an optimal balance between mechanical stability and controlled degradability, which implies exploring synthetic routes that enable dynamic and reversible bonds, as in the case of DA reactions, allowing adaptive hydrogels capable of responding to physiological signals and facilitating processes such as angiogenesis and tissue remodeling. Similarly, a thorough study of immunomodulatory strategies (such as stiffness modulation to favor M2 polarization and prevent chronic post-implant inflammation) is required as an indispensable prerequisite for safe medical applications [265].

Another key aspect is the scalability of these technologies and the safety of click processes, which must be addressed rigorously [266]. In this regard, future research should focus on more economical, sustainable, and adaptable bioorthogonal chemistry, integrating principles of green chemistry and advanced manufacturing [267]. Thus, it is clear that future investigations should contribute to the development of click-type reactions for the fabrication of hydrogels suitable for implant coatings and invasive surgical treatments; moreover, future research should aim to optimize these materials to establish a proper balance between cost and application of these technologies, which can contribute to more efficient therapies and treatment of various injuries and medical conditions [268].

The regulatory and/or legislative frameworks for biomaterial therapies and applications in regenerative medicine were extensively reviewed by Yoon et al. (2024), who prioritized regulatory frameworks for the use of biomaterials in tissue regeneration and for the delivery of drugs and biomolecules across various countries. For example, in the United States, the Regenerative Medicine Advanced Therapy (RMAT) designation is supported by the 21st Century Cures Act, and the minimum requirements for tissue-based treatments are regulated under 21 CFR Part 1271. In the European Union, advanced therapy products (ATMPs) are strictly regulated by Regulation (EC) No 1394/2007 [269].

## 7. Conclusions

Click-type reactions have transformed the synthesis of materials such as biocompatible hydrogels for medical applications through a versatile, efficient, and highly controllable approach. However, the expected impact of these technologies on medical applications is limited by several critical aspects: the presence of residual copper in the CuAAC mechanism, which generates inflammatory responses and ROS; the series of sequential steps that require alternative mechanisms such as SPAAC, which increase the operational costs for the synthesis of precursors for biorthogonal reactions in the functionalization of biomaterials; and the high costs of commercial precursors such as dibenzocyclooctyne-PEG_4_-maleimide (ideal for biorthogonal reactions via SPAAC), alkyne-PEG_4_-maleimide, SM(PEG)_24_ (PEGylated, long-chain SMCC cross-linker), DBCO-dPEG^®^_12_-MAL, precursors for Diels–Alder-type reactions: MAL-dPEG^®^_8_-acid, and for thiol/ene reactions: thiol-dPEG^®^_12_-acid. Furthermore, strict quality controls in manufacturing and functionality establish prohibitive conditions for the scalability of these technologies.

This work has demonstrated that the transition from chemical synthesis to clinical functionality of relevant biomaterials requires a comprehensive approach that integrates molecular mechanisms and low-cost synthesis alternatives and is supported by regulations and/or legislation that promote the industrial viability of these technologies. For example, the use of biomaterials such as hydrogels can be endorsed by Section 3033 of the 21st Century Cure Act, which regulates the use of drugs and materials in regenerative therapies, as well as the designation of medicines and materials as potential treatments for medical emergencies, and by European Union (EU) Regulation (EC) No 1394/2007, which covers products and activities in tissue engineering and cell therapies.

Furthermore, the most relevant findings highlight the influence of structural parameters, including crosslinking density, chitosan deacetylation degree, and molecular weight, as these factors determine stiffness, degradability, and interactions with the extracellular matrix. Furthermore, the incorporation of dynamic, reversible bonds is a promising strategy for mimicking soft-tissue plasticity and reducing the risk of chronic inflammation. In addition, it is crucial to evaluate all aspects of material cytotoxicity and reaction byproducts critically, and to combine immunomodulatory strategies that favor polarization toward the M2 phenotype, thereby promoting tissue regeneration. Future advances in hydrogels synthesized or functionalized via click reactions should contribute to global efforts to generate robust quantitative data (such as viscoelastic moduli, fracture stresses, and hydrolytic absorption and release kinetics of drugs/biomolecules), as well as preclinical studies that account for enzymatic variability across species/biomodels. Together, it will be essential to address the challenges of scalability and sustainability by developing safer, more economically viable bioorthogonal processes that enable the secure, cost-effective treatment of public health problems requiring highly invasive surgical interventions, thus ensuring health and well-being, as established by the Sustainable Development Goals (SDGs). SDG 3: Good Health and Well-being and SDG 9: Industry, Innovation and Infrastructure.

## Data Availability

The data presented in this study are openly available in the article.
